# Back-spliced RNA from retrotransposon binds to centromere and regulates centromeric chromatin loops in maize

**DOI:** 10.1371/journal.pbio.3000582

**Published:** 2020-01-29

**Authors:** Yalin Liu, Handong Su, Jing Zhang, Yang Liu, Chao Feng, Fangpu Han

**Affiliations:** 1 State Key Laboratory of Plant Cell and Chromosome Engineering, Institute of Genetics and Developmental Biology, Chinese Academy of Sciences, Beijing, China; 2 University of Chinese Academy of Sciences, Beijing, China; University of California Riverside, UNITED STATES

## Abstract

In most plants, centromeric DNA contains highly repetitive sequences, including tandem repeats and retrotransposons; however, the roles of these sequences in the structure and function of the centromere are unclear. Here, we found that multiple RNA sequences from centromeric retrotransposons (CRMs) were enriched in maize (*Zea mays*) centromeres, and back-spliced RNAs were generated from CRM1. We identified 3 types of CRM1-derived circular RNAs with the same back-splicing site based on the back-spliced sequences. These circular RNAs bound to the centromere through R-loops. Two R-loop sites inside a single circular RNA promoted the formation of chromatin loops in CRM1 regions. When RNA interference (RNAi) was used to target the back-splicing site of the circular CRM1 RNAs, the levels of R-loops and chromatin loops formed by these circular RNAs decreased, while the levels of R-loops produced by linear RNAs with similar binding sites increased. Linear RNAs with only one R-loop site could not promote chromatin loop formation. Higher levels of R-loops and lower levels of chromatin loops in the CRM1 regions of RNAi plants led to a reduced localization of the centromeric H3 variant (CENH3). Our work reveals centromeric chromatin organization by circular CRM1 RNAs via R-loops and chromatin loops, which suggested that CRM1 elements might help build a suitable chromatin environment during centromere evolution. These results highlight that R-loops are integral components of centromeric chromatin and proper centromere structure is essential for CENH3 localization.

## Abstract 2

Circular retrotransposon RNAs generated by back-splicing bind to chromatin and mediate dynamic regulation of R-loops and centromeric chromatin loops in maize, driving centromeric chromatin organization and CENH3 localization.

## Introduction

Centromeres are located in the primary constriction of chromosomes and enable the correct separation of chromosomes during mitosis and meiosis. Active centromeres are marked by a centromeric specific histone H3 variant, named CENH3 in plants [1,2] and centromeric protein A (CENP-A) in animals [3,4]. In most eukaryotes, centromeric DNA contains hierarchical arrays of highly repetitive sequences; in humans, centromeric repeats consist mostly of simple tandem repeats [5,6], whereas in plants, multiple retrotransposons are intermingled with tandem repeats in the centromeric regions, producing centromeres several megabases in size [7]. In maize (*Z*. *mays*), several centromeres (Cen2, Cen5, and Cen10) have been well sequenced [8,9], and maize centromeric specific DNA contains a 156-bp tandem repeat (CentC) and centromeric retrotransposons (CRMs) [10].

There are 4 kinds of CRMs in maize, CRM1–CRM4 [11,12], with CRM1 and CRM2 constituting the majority of sequences in maize centromeric regions [8]. In the functional maize centromeric region—identified using anti-CENH3 chromatin immunoprecipitation following high-throughput sequencing (ChIP-seq)—the distribution of CRM2 coincides with the deposition sites of CENH3 nucleosomes [8,13]. Fewer interactions with CENH3 nucleosomes have been detected for CRM1 than for CRM2, but more occur for CRM3 [8]. CRM4 elements are no longer active in maize, and they are not located at the core centromeric regions [11]. Fluorescence in situ hybridization (FISH) experiments showed that the distributions of the CRM1 and CRM2 elements overlap in the centromeric regions during metaphase but that the CRM2 elements tend to localize to the outer centromeric region, while CRM1 elements localize more prominently at the inner centromeric region [8,11,14]. CRM1 has the largest number of nucleotides (nt) comprising full-length elements among the 4 CRM subfamilies and is the most active CRM element in maize centromeres [11]. The expansion of the maize centromere sizes during evolution is related to a shift of CRM1 [8]. The roles of the CRM elements in centromeric function may differ; therefore, the function of the CRM1 element in the centromeres needs to be explored.

The complex composition and arrangement of centromeric repeats in most species make it difficult to fill centromeric gaps during genome assembly and recognize their roles in centromere organization [15]. Many studies have focused on the interactions between centromeric DNA sequences and CENH3 localization. In artificial human chromosomes, centromere specification can be partly determined by the DNA sequence [16], as centromeric chromatin and pericentromeric heterochromatin in humans depend on both DNA sequences and epigenetic factors [17]. De novo centromeres can form at ectopic regions of the chromosomes and inactive centromeres without CENH3 localization, demonstrating that centromeric specific DNA itself is not completely necessary or sufficient for centromere formation and maintenance [18–21]. In budding yeast (*Saccharomyces cerevisiae*), the topological structure of the centromere can be induced by the sequence of the single centromeric nucleosome [22]. During evolution, the expansion of centromeric sequences resulted in stronger centromeres with increased competitiveness during meiosis [23]. Not all centromeric DNA sequences are associated with the CENH3 nucleosomes directly; thus, some of the centromeric DNA may induce the formation of centromeric chromatin to create a stable environment for efficient CENH3 localization.

Centromere transcription processes have been reported to play roles in centromere assembly [24,25]. The inhibition of RNA Polymerase II (RNAPII) can lead to the unloading of CENP-A nucleosomes in mammalian centromeres [26], while chromatin remodelers such as the Facilitates Chromatin Transcription (FACT) complex can be involved in centromere transcription and CENP-A deposition in flies (*Drosophila melanogaster*) [27]. RNAPII-dependent transcription can drive Shugoshin from the kinetochore to the inner centromeric chromatin during metaphase in human cells [28]. Centromeric RNA is also essential for centromere function [29,30], and centromeric satellite RNA is known to be a key component for the recruitment of centromere-specific nucleoproteins at the nucleolus and mitotic centromere [31]. Specific satellite RNAs have been identified in human and fly centromeres, with decreased RNA levels leading to abnormal centromere function and incorrect chromosome behaviors [32,33]. Furthermore, the RNA produced from the active α-satellite arrays in human centromeres is essential for CENP-A loading [17], and transcripts from the LINE-1 (L1) retrotransposon are an essential structural and functional component of neocentromeric chromatin [34]. In budding yeast, centromeric RNA can function *in trans* to regulate centromere activity, with too much or too little centromeric RNA leading to a centromere malfunction [35]. Long noncoding RNA (lncRNA) from the centromere of *Xenopus laevis* is required for the localization and activation of the chromosomal passenger complex in the inner centromeric regions [36]. In maize, RNA from different regions of the CRM2 element has been detected using anti-CENH3 RNA immunoprecipitation (RIP) [37]. Previous studies have emphasized the importance of the CRM2 element in maize centromeric function, although RNAs from the other CRM elements have been less well studied. All of these well-characterized centromeric RNAs were amplified or detected based on well-known centromeric specific DNA sequences; thus, we do not currently have a complete view of centromeric RNA and the potentially spliced sequences of these RNAs. A new method has been developed to systemically identify centromeric RNAs [38]. Centromeric RNA may interact with chromatin-binding factors, affect nucleosome assembly, and regulate chromatin structure in the centromere, but the detailed mechanisms involved in these processes are unknown.

R-loops containing RNA:DNA hybrids and single-strand DNA (ssDNA) can also influence chromatin activity [39]. In yeast, RNA:DNA hybrids play an important role in the formation of heterochromatin and in RNA interference (RNAi) [40]. Phosphorylation of serine 10 in histone H3 (H3-Ser10) in the pericentromeric heterochromatin is also related to R-loops in budding yeast [41,42]. Genome-wide R-loops with multiple chromatin modifications have been found in *Arabidopsis thaliana* [43]. R-loops can recruit their reader (growth arrest and DNA damage protein 45A [GADD45A]) to promote the demethylation pathway at promoter cytosine-phosphate-guanine (CpG) islands in human cells [44]. Chromatin-binding RNAs can influence the structure and activity of chromatin [45,46]; for example, lncRNA from *Flowering Locus C* in *Arabidopsis* takes part in the formation of chromatin loops to regulate gene expression [47]. However, whether centromeric RNA interacts with DNA or proteins in the centromeric region to regulate centromeric chromatin requires further study.

Circular RNA is formed by fusing the downstream 3′-splice site and the upstream 5′-splice site of a pre-messenger RNA, through the process of back splicing [48]. For circular RNAs produced from exons, the pairing of inverted *Arthrobacter luteus* (Alu) repeats in the flanking introns can lead to exon circularization [49]. In plants, whole-genome analyses of circular RNA have been performed, and multiple intron- or exon-derived circular RNAs with noncanonical GT/AG signals have been found in rice (*Oryza sativa*) and polyploid cotton (*Gossypium* sp.) [50,51]. Many studies have shown that circular RNAs can regulate the mRNA levels of target genes [52–54]; for example, in *Arabidopsis*, circular RNA from exons was shown to bind to the target gene through the formation of R-loops, resulting in depressed gene transcription [55]. Unlike circular RNAs produced from coding genes, the production of circular RNAs from retrotransposons has not been reported, and their chromatin-related functions require elucidation.

This study addresses the interaction between CRMs and centromere structure and function. Three types of circular RNAs are produced from CRM1 in maize, each with different sizes but the same back-splicing site. These circular RNAs can bind to centromeric chromatin through R-loops. The two R-loop sites inside a single circular RNA promote the formation of chromatin loops. When RNAi was performed to target the back-splicing site of the circular RNAs, the levels of R-loops and chromatin loops formed by the circular RNAs decreased; however, the R-loops of the two kinds of linear RNAs with similar R-loop formation sites increased. The high levels of R-loops and low levels of chromatin loops in the CRM1 regions led to a reduced CENH3 localization in the RNAi plants. The process of back splicing in retrotransposons was also found to be conserved in numerous crop species.

## Results

### Specific back-spliced RNA from CRM1 binds to maize centromeres

**Fig 1:**
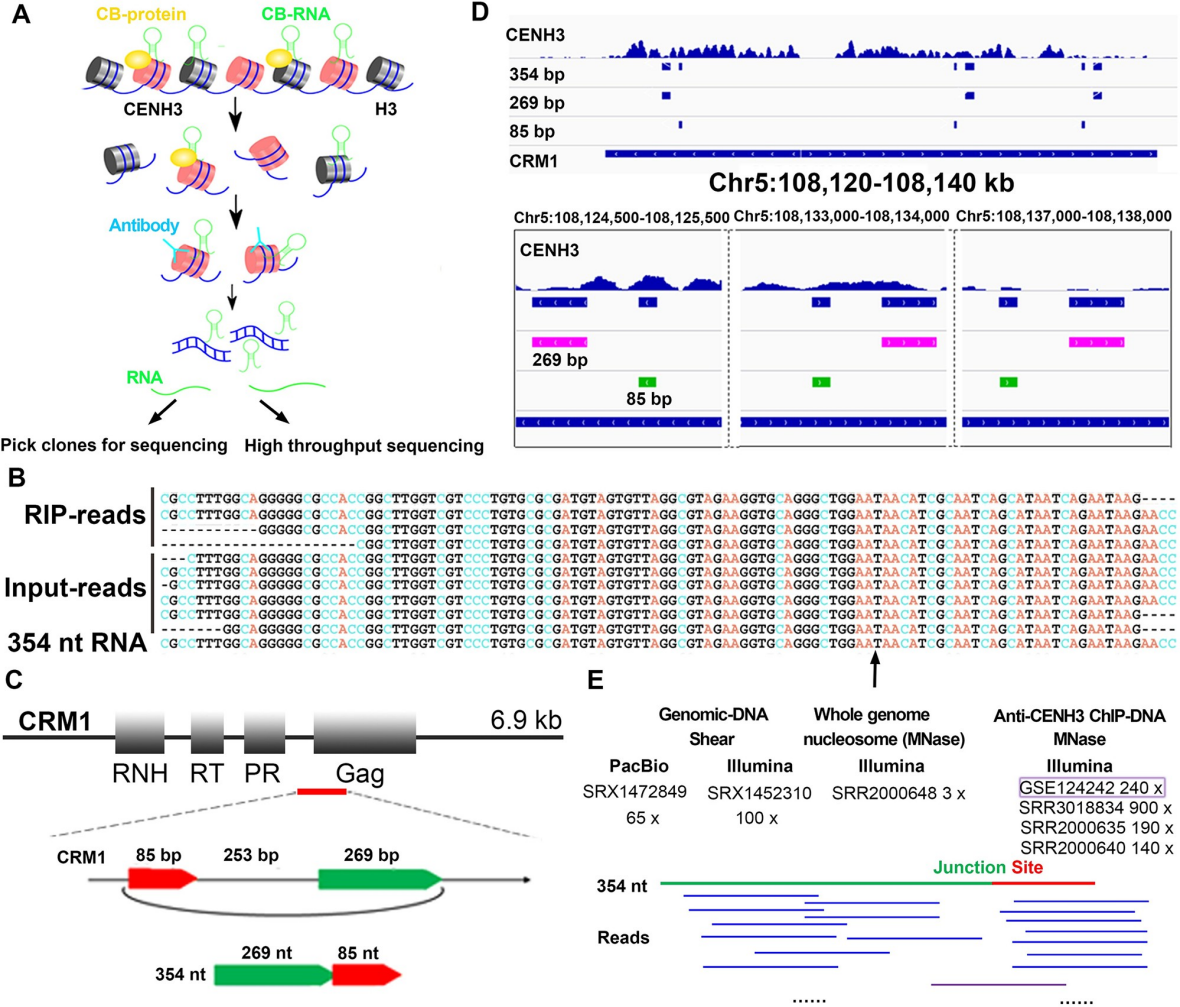
Back-spliced CRM1 RNA in the maize centromere. (A) Procedure for anti-CENH3 RIP and subsequent high-throughput sequencing and cDNA library screening. (B) BLAST results of the back-spliced CRM1 reads from the anti-CENH3 RIP-seq, input-seq data, and the 354-nt RNA from the anti-CENH3 RIP cDNA library. The arrow shows the back-splicing site. (C) The location of the 607-bp combined sequence in CRM1, and the back-spliced form of the 354-nt RNA. The red line represents the 607-bp sequence. (D) Distribution of the 354-bp sequence on CRM1. The first track of each panel represents the centromeric region indicated by CENH3 enrichment, and the peak height represents the RPM value (0–1). The other 4 tracks represent the distributions of the 354-bp, 269-bp, 85-bp, and CRM1 sequences along a specific region of cen5. The lower panel shows a detailed version of the information displayed in the upper panel. The arrows inside the rectangular bars represent the directions of the sequences. (E) The public raw genome sequencing data (including Pacbio [65×] and Illumina [100×] reads) and 4 anti-CENH3 ChIP-seq datasets from B73 (including 1 generated in this study and 3 from public resources), together with 1 input-seq dataset, were mapped to the assumed 354-bp DNA. Only one read from anti-CENH3 ChIP-seq dataset was matched to the region containing the back-spliced junction site (purple line). All the other reads show no covering the back-spliced junction site. The data underlying this figure can be found in the GEO with accession numbers GSE124242, SRR3018834, SRR2000635, SRR2000640, SRR2000648, SRX1472849, and SRX1452310 and on Github (https://github.com/sxx-ying/maize-centromere-circRNA). CB, chromatin binding; CENH3, centromeric H3 variant; ChIP-seq, chromatin immunoprecipitation following high-throughput sequencing; Chr, chromosome; CRM, centromeric retrotransposon; Gag, gag protein; GEO, Gene Expression Omnibus; input-seq, input sequencing; nt, nucleotides; PR, protease; RIP, RNA immunoprecipitation; RIP-seq, RIP sequencing; RNH, RNase H; RPM, reads per million; RT, reverse transcriptase.

For detailed analysis of centromeric RNA, we used anti-CENH3 RIP in the maize inbred line B73 to capture RNA bound to the centromeric regions, using both high-throughput sequencing and cDNA library screening methods (Fig 1A). Centromeric RNA can be generated from centromeric specific DNA according to previous studies [17,29–33]. CRM elements are well-known CRMs, which constitute the majority of the sequences in maize centromeres. We therefore focused on RNA produced from the CRM elements in this work.

The commonly used mapping programs for high-throughput sequence analyses, including tophat2, hisat2, bwa, bowtie2 and STAR, could not handle the repeat sequences well; therefore, extensively used BLAST software was performed to detect all the reads mapped to the centromeric repeats (S1A Fig). Abundant reads from anti-CENH3 RIP sequencing (RIP-seq) data were generated from the centromeric repeats, including CRM1, CRM2, CentA (CentA was the first CRM element identified in maize, and CRM3 is related to the nonautonomous CentA [11]), and CentC (S1B Fig). Reads from 2 active genes in the centromeric regions (Zm00001d030471 and Zm00001d004256) were enriched in the RIP-seq data (S1B Fig); however, 2 unexpressed genes (Zm00001d004248 and Zm00001d030471) located in centromeric regions near CRM1 elements displayed no enrichment (S1B Fig). The high enrichment of reads from the centromeric sequences indicated that the RIP-seq protocol worked well.

The reads containing long terminal repeat (LTR) sequences were classified as LTR reads. Aside from these, the reads aligned to only 1 CRM element site were identified as nonspliced reads, while those aligned to 2 separate sites were referred to as spliced reads. LTR and nonspliced reads accounted for almost all of the reads from the CRM elements and were distributed across the CRM regions (Table 1). A few spliced reads were identified for each CRM element (Table 1). Among all the reads mapped to CRM1, back-spliced reads were identified from both the RIP data and the input data (Table 1). Numerous normally spliced RIP reads were found to map to the CRM3 element, with the least being mapped to the CRM2 element (Table 1). Back-spliced reads were only found in the CRM1 elements.

**Table 1:** The number of merged fragments from anti-CENH3 RIP mapped to the CRM elements.

		anti-CENH3 RIP L5_A012	Input L5_A010
Reads mapped to CRM		Number of reads	Number of reads
	Nonspliced reads	8,969	674
**CRM1**	Spliced reads	7 back-spliced 5 normal-spliced	7 back-spliced
	LTR reads	8,547	336
	Nonspliced reads	9,027	371
**CRM2**	Spliced reads	2 normal-spliced	0
	LTR reads	15,104	254
	Nonspliced reads	6,187	354
**CRM3**	Spliced reads	125 normal-spliced	1 normal-spliced
	LTR reads	1,989	64
**Total merged-fragments**		26,400,191	13,603,923

**Abbreviations:** CENH3, centromeric H3 variant; CRM, centromeric retrotransposon; LTR, long terminal repeat; RIP, RNA immunoprecipitation

The RNAs from nonspliced and LTR reads were directly transcribed from the CRM elements, which could be confirmed easily according to their DNA sequences. Many previous studies have explored the functions of the direct transcripts from the centromeres [29–36]. CRM2 RNA was found in the same way [37]. Back-spliced RNAs from CRMs have not previously been reported, and the roles of CRM1 in centromeric function are confusing. We therefore focused on the roles of back-spliced RNA from CRM1 in maize centromeres.

Among all the back-spliced reads mapped to CRM1 in the RIP and input data, 4 and 6 reads were found to have the same back-splicing sites, suggesting that these reads may be derived from the same RNA (Fig 1B). This back-spliced RNA from CRM1 may be not located specifically at the CENH3-nucleosomes–occupied subregions, since the input data also contained similar reads. CRM1 elements are distributed along the whole centromere but are not perfectly associated with CENH3 nucleosomes; therefore, it is possible that the back-spliced CRM1 RNA can bind to the centromere without being selected to interact with the CENH3 nucleosomes. This may be one of the reasons for the low ratio of back-spliced CRM1 reads in the anti-CENH3 RIP-seq data.

Despite sharing a back-splicing site, the lengths of these 10 reads from CRM1 were different; we only obtained an RNA sequence less than 250 nt in size after merging all the reads, which may not cover the whole length of the original back-spliced RNA (Fig 1B). We then screened the anti-CENH3 RIP cDNA library to look for longer forms of the back-spliced RNA (Fig 1A). After sequencing 2,000 clones, we identified a 354-nt RNA sequence (Fig 1B). This sequence appeared many times in the later screen and was derived from CRM1, as was demonstrated by aligning it to the annotated maize centromere bacterial artificial chromosome (BAC) ZM16H10 [56]. The 354-nt RNA had the same back-splicing site as the 10 back-spliced reads from CRM1, and the length of this sequence covered these 10 reads (Fig 1B). The 354-nt sequence was only detected after reverse transcription, indicating that it existed as an RNA (S1D Fig). This 354-nt RNA was therefore chosen to represent the 10 back-spliced CRM1 reads that were identified from the anti-CENH3 RIP-seq and input data. The 354-bp clone had signals at the centromeric regions, as confirmed using DNA-FISH (S1C Fig). The distribution of the 354-bp clone sequence was coincident with CRM1 elements along the centromere in the genome (S1E and S1F Fig).

Two discontinuous portions of the 354-nt sequence, which we refer to as the 269-nt and 85-nt sequences, were exactly matched to the CRM1 element (Fig 1C). The downstream 3′-splice site of the 269-nt sequence was fused to the upstream 5′-splice site of the 85-nt sequence in the 354-nt RNA, suggesting that the 354-nt sequence is a back-spliced RNA (Fig 1C). The 85-bp and 269-bp sequences were separated by a 253-bp sequence to generate a 607-bp sequence (Fig 1C), spanning from the proteinase-coding region to the adjacent intergenic region of CRM1, which is not a typical exon or intron site (Fig 1C). A search of the entire centromere showed that all the copies of the 354-bp clone sequence were divided into 269-bp and 85-bp sequences in the CRM1 elements (Fig 1D). The regions containing the 269-bp and 253-bp sequences were associated with CENH3 nucleosomes (S1G Fig).

In order to exclude the possibility of a genomic origin for the 354-bp DNA sequence, we checked the maize B73 RefGen_v4 genome, which was assembled using single-molecule real-time sequencing, and the assumed 354-bp sequence was not detected. Additionally, we mapped the raw reads from Pacbio (65**×**) and Illumina (100**×**) whole-genome sequencing data to the assumed 354-bp DNA, and no reads in the public datasets were matched to the back-splicing junction site (Fig 1E). Furthermore, 4 anti-CENH3 ChIP-seq datasets from B73, including 1 from our lab and 3 from public resources (centromere coverages range from 140**×** to 900**×**), together with 1 input sequencing (input-seq) dataset (genome coverage 3**×**), were BLAST-searched with the unassigned 354-bp DNA sequence as the template. No reads from the 3 public datasets and the input-seq dataset were found to match the region containing the back-splicing site. Only one read from our anti-CENH3 ChIP-seq dataset was mapped to the 354-bp sequence containing the back-splicing site (Fig 1E), which may have been derived from residual chromatin-binding RNA in the ChIP sample. These results suggested that there is no 354-bp DNA sequence in the genome. Taken together, we identified a 354-nt back-spliced RNA derived from CRM1, which binds to centromeric regions.

### Detection of full-length circular RNAs from CRM1

**Fig 2:**
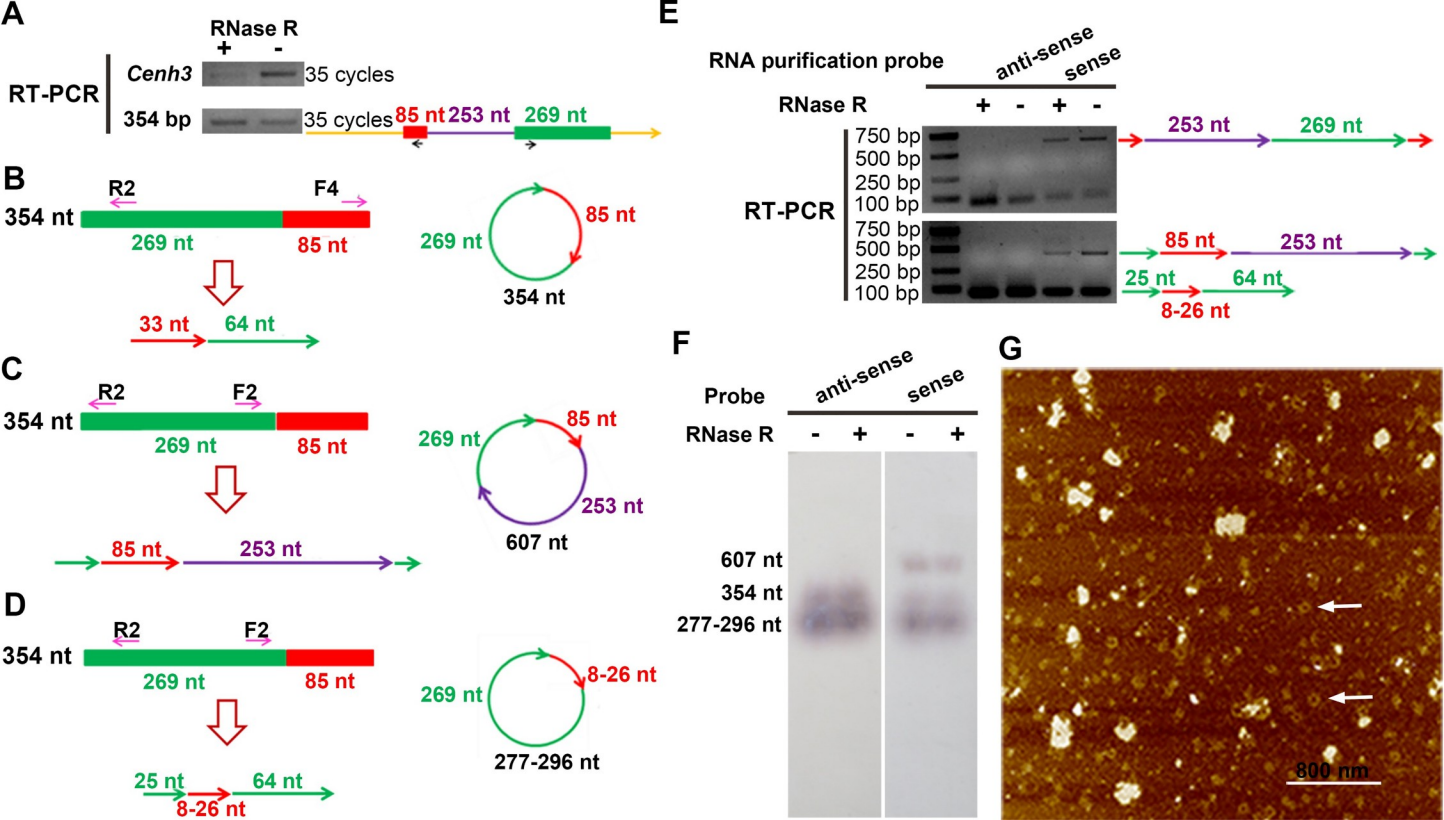
Detection of the full-length circular CRM1 RNAs. (A) The 354-nt RNA was stable after an RNase R treatment. *Cenh3* mRNA was used as a linear RNA control. The black arrows in the right panel show the positions of primers used. (B) Divergent primers F4+R2 were used to detect the existence of the 354-nt circular RNA (left panel). The right panel shows the form of the 354-nt circular RNA. (C) Divergent primers F2+R2 targeting the 269-nt sequence confirmed the existence of the 607-nt circular RNA (left panel). The right panel shows the form of the 607-nt circular RNA. (D) Divergent primers F2+R2 also confirmed the existence of 277- to 296-nt circular RNAs (left panel). The right panel shows the form of the 277- to 296-nt circular RNA. In (B–D), the upper models show the position of primers on the 354-nt sequences, while the lower models show the amplified sequences. The right schematic diagrams show the circular RNA with corresponding sizes. (E) Divergent PCR showed that the 607-nt and 277- to 296-nt RNAs were stable after RNase R treatment. The right panel shows the compositions of the amplified sequences mentioned in Figs 2C, 2D and S2E. (F) A northern blot was performed using B73 RNA purified with biotinylated antisense or sense oligos, then detected using digoxin-labeled 25-bp antisense or sense probes. The probe was located in the 269-nt region. The RNA was run in 3% denaturing formaldehyde agarose gel. (G) AFM image of the circular CRM1 RNAs. The RNAs were purified by biotinylated sense oligo, followed by RNAse R treatment. The white arrows indicate the circular RNA. The scale bar is 800 nm. In (A–E), the yellow, red, green, and purple arrows represent the directions of the sequences. The short arrows under or above the sequences represent the positions of the primers. The data underlying this figure can be found in S1 Raw Images. AFM, atomic force microscopy; CRM, centromeric retrotransposon; nt, nucleotides; RT-PCR, reverse transcription PCR.

Because the 354-nt RNA was produced from CRM1 by back splicing, we next investigated whether it was a circular form and sought to determine its full-length sequence. The 354-nt RNA was stable after RNase R treatment of poly(A)-RNA/rRNA-RNA (Fig 2A), which demonstrated the circular nature of its form. We then designed divergent primers to confirm the full-length sequence of the 354-nt RNA. We found that the PCR template obtained using cDNA generated from the total RNA could not be efficiently amplified; therefore, templates from RNA purified with 354-bp biotinylated ssDNA probes were used instead. Five pairs of divergent primers were utilized.

Two divergent primers (F4+R2 and F3+R3) for the 354-nt RNA were used to show the direct fusion of the 3′-splice site in the 85-nt sequence to the 5′-splice site in the 269-nt sequence (Figs 2B, S2A, S2B, and S2C), demonstrating that the 354-nt sequence was an intact circular RNA (Fig 2B, right part). During amplification, we found additional sequences related to the 354-nt RNA. Two divergent primers (F1+R1 and F2+R2) were used to reveal that the 253-nt sequence was located between the 85-nt and the 269-nt sequences in the complete RNA molecule (Figs 2C, S2D, S2E and S2F). These results indicated that an integrated 607-nt circular RNA was produced from the 607-bp DNA of CRM1 and had the same back-splicing site as the 354-nt circular RNA (Fig 2C, right part).

Additionally, the primers F2+R2 could be used to amplify a type of shorter sequence with an 8- to 26-nt fragment of the 85-nt sequence fused to the 5′-splice site in the 269-nt sequence (Figs 2D and S2G). Furthermore, another divergent primer pair (F2+R3) amplified similar sequences of 17 to 27 nt from the 85-nt sequence (S2H and S2I Fig). These results confirmed the existence of circular RNAs ranging from 277 to 296 nt in length that exhibited the same back-splicing site as the 354-nt and 607-nt circular RNAs (Fig 2D, right part). More variants similar to the 277- to 296-nt circular RNAs may exist. Although we used 2 pairs of primers, the clones that we obtained for sequencing were limited. Both the 607-nt and 277- to 296-nt RNAs were stable after the RNase R treatment, further confirming that they were circular RNAs (Fig 2E). The 607-nt circular RNA was produced from the sense strand, while the 277- to 296-nt circular RNAs were produced from both the antisense and sense strands (Fig 2E).

To confirm the full lengths of these circular RNAs, we performed northern blotting using RNAs purified with 354-bp biotinylated sense and antisense oligos. Digoxin-labeled 25-bp ssDNA probes from the 269-bp sequence were used for northern blotting. The 354-nt and 277- to 296-nt circular RNAs could be detected using both the sense and antisense probes; however, the 607-nt circular RNAs could only be detected using the sense probe (Fig 2F). The RNase R treatment had no obvious effects on any of the circular RNAs (Fig 2F). In order to determine the existence of the circular CRM1 RNAs with low abundance, we checked these circular RNAs using atomic force microscopy (AFM). The biotinylated sense oligos covering the back-splicing site were used to capture related RNAs from the total RNA of B73 leaves. The purified RNAs were treated with RNase R to enrich circular RNAs and then were imaged with AFM. The circular RNAs were clearly detected, and the lengths of these molecules measured by the standard scale were varied from 280 nt to 700 nt, which is consistent with the PCR and northern blotting results. (Fig 2G). The average height of these circular RNAs was about 600 pm, and the widths and circumferences of these molecules ranged from 30 to 60 nm and from 100 to 200 nm, respectively (S2J Fig). The ratio of circular molecules’ number to larger complexes per surface scanned was approximately 0.32, which was consistent with our initial interpretation that these molecules are really quite low in number. Based on these results, we inferred that the CRM1 element produces 3 types of circular RNAs with the same back-splicing site.

### Circular CRM1 RNAs induce chromatin loops in the centromeres

**Fig 3:**
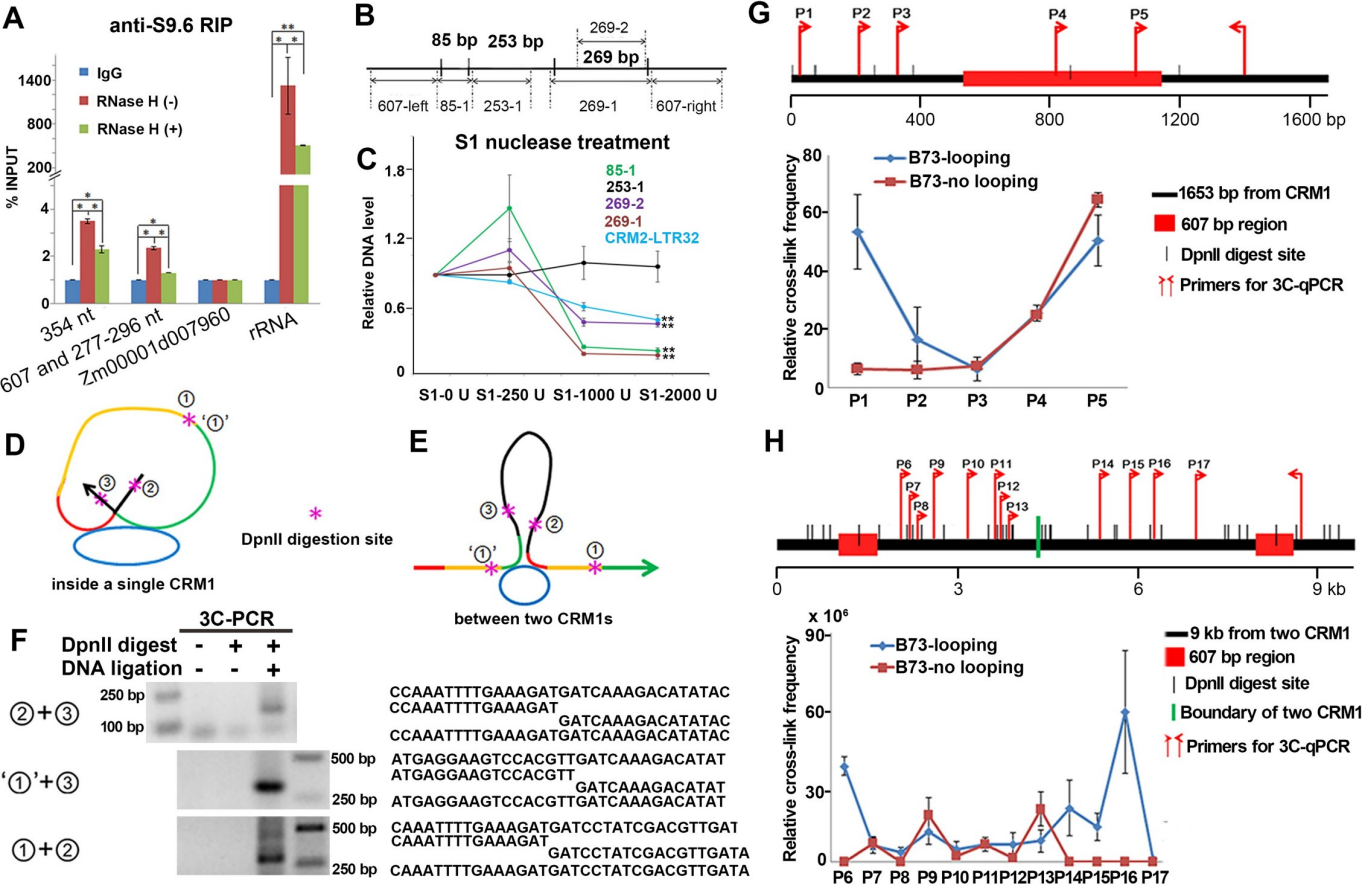
Circular CRM1 RNAs induce chromatin loops in the centromere. (A) Anti-S9.6 RIP-qPCR was used to confirm the R-loop formation by 354-, 607-, and 277- to 296-nt circular CRM1 RNAs. Zm00001d007960 RNA was used as a negative control, and rRNA was used as a positive control. Chromatin-binding RNA was used for RIP. Actin was used as an internal reference gene. (B) Regions chosen for detecting the ssDNA sites are marked as 85–1, 253–1, 269–1, and 269–2. (C) ssDNA sites in CRM1 were checked using an S1 nuclease treatment of the nuclear DNA. DNA with no S1 nuclease treatment was used as a control template. The 607-left sequence was used as an internal reference gene. (D and E) Potential chromatin loops were induced by circular RNA inside a single CRM1 element (D) and between two CRM1 elements (E). Red, green, and yellow lines represent the 85-, 269-, and 253-bp regions, respectively. Black lines represent sequences on the left side of the 85-bp sequence and the right side of the 269-bp sequence. The blue ovals represent circular CRM1 RNAs. ①, ‘①’, ②, and ③ represent the broken ends on the two sides of the 253-bp sequence, the left side of the 85-bp sequence, and the right side of the 269-bp sequence. (F) 3C-PCR confirms the potential ligations of chromatin loops after *DpnII* digestion. The left panel shows the PCR results in the undigested, unligated samples and 3C samples under potential ligation forms. The right panel shows the sequences from the bands on the left, including the expected sequences, the first and the second part of the expected sequences, and the amplified sequences. (G and H) 3C-qPCR shows chromatin interactions inside a single CRM1 element (G) and between two CRM1 elements (H). The interaction frequencies between two *DpnII*-digested fragments were normalized to the 3C control template from the digested and ligated centromeric BAC clone and an internal reference gene, *SAM*. In (A), (C), (G), and (H), the columns and error bars represent the relative value and standard error of the means (*n* = 3). In (A) and (C), the *P* values were determined using a Student *t* test: *P < 0.05, **P < 0.01. The data underlying this figure can be found in S1 Data and S1 Raw Images. 3C, chromatin conformation capture; BAC, bacterial artificial chromosome; CRM, centromeric retrotransposon; IgG, Immunoglobulin G; nt, nucleotides; qPCR, quantitative PCR; RIP, RNA immunoprecipitation; ssDNA, single-strand DNA.

How do the circular RNAs bind to the centromeric chromatin? First, we wondered whether the circular RNAs bind to the centromeres through R-loops. RNase H was used to treat B73 chromatin-binding RNAs to detect the formation of RNA:DNA hybrids by these circular CRM1 RNAs (S3A Fig). The 354-, 607-, and 277- to 296-nt circular RNAs were sensitive to the RNase H treatment (S3A Fig). The RIP was performed using the RNA:DNA hybrid-specific S9.6 antibody to confirm the formation of R-loops. According to the genome-mapping results of the anti-S9.6 DNA-RNA immunoprecipitation sequencing (DRIP-seq) data from *Arabidopsis*, the ribosomal DNA (rDNA) regions are highly enriched with R-loops, and the gene AT1G24510—which encodes a chaperonin-60/T-complex protein (TCP-1/cpn60) chaperonin family protein—showed no R-loop formation [43]. The conserved rRNA region including a 5.8S rRNA sequence in maize was therefore used as a positive control for the anti-S9.6 RIP-quantitative PCR (qPCR), while the maize homolog of AT1G24510, Zm00001d007960, was used as a negative control. All 3 types of circular CRM1 RNAs were enriched in the anti-S9.6 immunoprecipitated RNA sample and were sensitive to RNase H treatment (Fig 3A). These results suggest that R-loops were formed by these circular RNAs at the centromeric regions.

The R-loop formation sites of these circular RNAs could adopt different forms, such as one circular RNA binding to only one single site (case 1), multiple circular RNAs binding to several sites inside one CRM1 element (case 2), or one circular RNA binding to two nearby sites simultaneously (case 3) (S3B Fig). Because both RNA:DNA hybrids and ssDNA exist in the R-loop regions, S1 nuclease was used to treat the B73 nuclear DNA to detect the ssDNA sites. Both the regions containing the 85-bp and 269-bp sequences were sensitive to S1 nuclease treatment, while the intermediate 253-bp region was not (Fig 3B and 3C). This result showed that R-loops can be formed both at the 85-bp and 269-bp regions, but not at the intermediate 253-bp region. The LTR region of the CRM2 element also exhibited a high sensitivity to S1 nuclease treatment (Fig 3C). Next, T7 endonuclease I was used to cleave non-perfectly matched DNA to check the R-loop regions. B73 nuclear DNA was treated with T7 endonuclease I, and the fragments were then ligated by T4 DNA ligase after filling in the ends. Sequences shorter than the original genomic sequences were identified using primers 1 and 3 (S3C Fig). These short sequences had lost the 57-bp sequence in the 85-bp region and lack the entire 253- and 269-bp regions (S3D Fig). This result suggested that there are 2 simultaneous R-loop sites inside one CRM1 element, at the 85- and 269-bp regions; therefore, the R-loop formation sites adopt forms described by case 2 or 3 (S3B Fig).

In case 3, circular RNA can bind to the 85- and 269-bp regions at the same time; thus, chromatin loops can be induced both inside a single CRM1 element and between 2 CRM1 elements (Fig 3D and 3E). However, the binding of circular RNA at 2 sites does not induce changes in chromatin conformation in case 2 (S3B Fig). Chromatin conformation capture (3C) was performed in B73 using a *DpnII* restriction endonuclease digestion to check whether chromatin loops were formed in CRM1 regions (Figs 3D, 3E and S3E). After a *DpnII* digestion, there were 5 potential ligations, designated ①+②, ①+③, ②+③, ‘①’+②, and ‘①’+③ (Fig 3E). Three of these, ②+③, ‘①’+②, and ‘①’+③, were detected only in the 3C sample and not in undigested and unligated samples (Fig 3F), indicating that chromatin loops were formed. These results suggest that chromatin loops in the CRM1 regions may be induced by circular RNA, as illustrated by case 3 (Figs 3D, 3E and S3B).

Chromatin interactions in the regions containing R-loop sites were also checked by 3C-qPCR in B73. The interaction frequency between 2 *DpnII* digestion-induced fragments was normalized to the 3C control template combining the digested and ligated centromeric BAC clone (ZMMBBb0497C16) containing the CRM1 elements and the internal reference gene *SAM* (encoding *S-adenosyl-methionine decarboxylase*, which maintains similar conformations in different tissues [57]). High cross-linking frequencies were observed both within a 1.6-kb CRM1 region (Fig 3G) and between 2 CRM1 elements (Fig 3H). The chromatin loops inside the single CRM1 element were indicated by high cross-linking frequencies at the fragments containing primers P1 and P2 (Fig 3G), while large chromatin loops were formed along the 2 CRM1 elements (Fig 3H). High chromatin interaction frequencies were exhibited at fragments containing primers P6, P14, P15, and P16 (Fig 3H). In the no-looping control, the cross-linking frequencies between 2 CRM1 elements did not increase as the distance between the two primers decreased (Fig 3H). Such results may be explained by the presence of fragmented CRM1 elements and the incomplete insertion of CRM1 sequences into the genome. The chromatin interaction frequencies between the 2 CRM1 elements are much higher than those within a single CRM1, suggesting that large-scale loops are abundant in the CRM1 elements (Fig 3G and 3H).

According to the anti-CENH3 RIP data, the amount of nonspliced RNA from the CRM elements is much larger than that of the back-spliced RNA at the centromeric regions (Table 1). To distinguish the roles of circular CRM1 RNAs and related nonspliced RNAs in the formation of chromatin loops, we chose 3 kinds of nonspliced RNAs from CRM1. These RNAs, containing the 85-nt sequence (RNA-85), the 269-nt sequence (RNA-269), or both (RNA-85+269), were sensitive to RNase R treatments, indicating that they were linear (S3F Fig). These linear CRM1 RNAs also bound to the centromere through R-loops, as confirmed by the results of the anti-S9.6 RIP (S3G Fig). Both CRM1 circular RNAs and linear RNAs formed R-loops, which seem to be integral components of centromeric chromatin.

Anti-S9.6 RIP of circular and linear CRM1 RNAs was conducted using chromatin-binding RNA samples (Figs 3A and S3G), which differed in their proportions of chromatin binding (S3H Fig). Approximately 14% of the 354-, 607-, and 277- to 296-nt circular RNAs bound to chromatin, while approximately half of the RNA-85 and RNA-269 sequences bound to chromatin (S3H Fig). These results suggest that only a small portion of circular CRM1 RNAs bind to chromatin to form R-loops and that the proportion of R-loops in linear CRM1 RNAs is higher than that of the circular CRM1 RNAs in B73. The influence of linear RNA on chromatin loop formation therefore requires further investigation.

### Decreased chromatin loops and CENH3 localization in the CRM1 regions of RNAi plants

**Fig 4:**
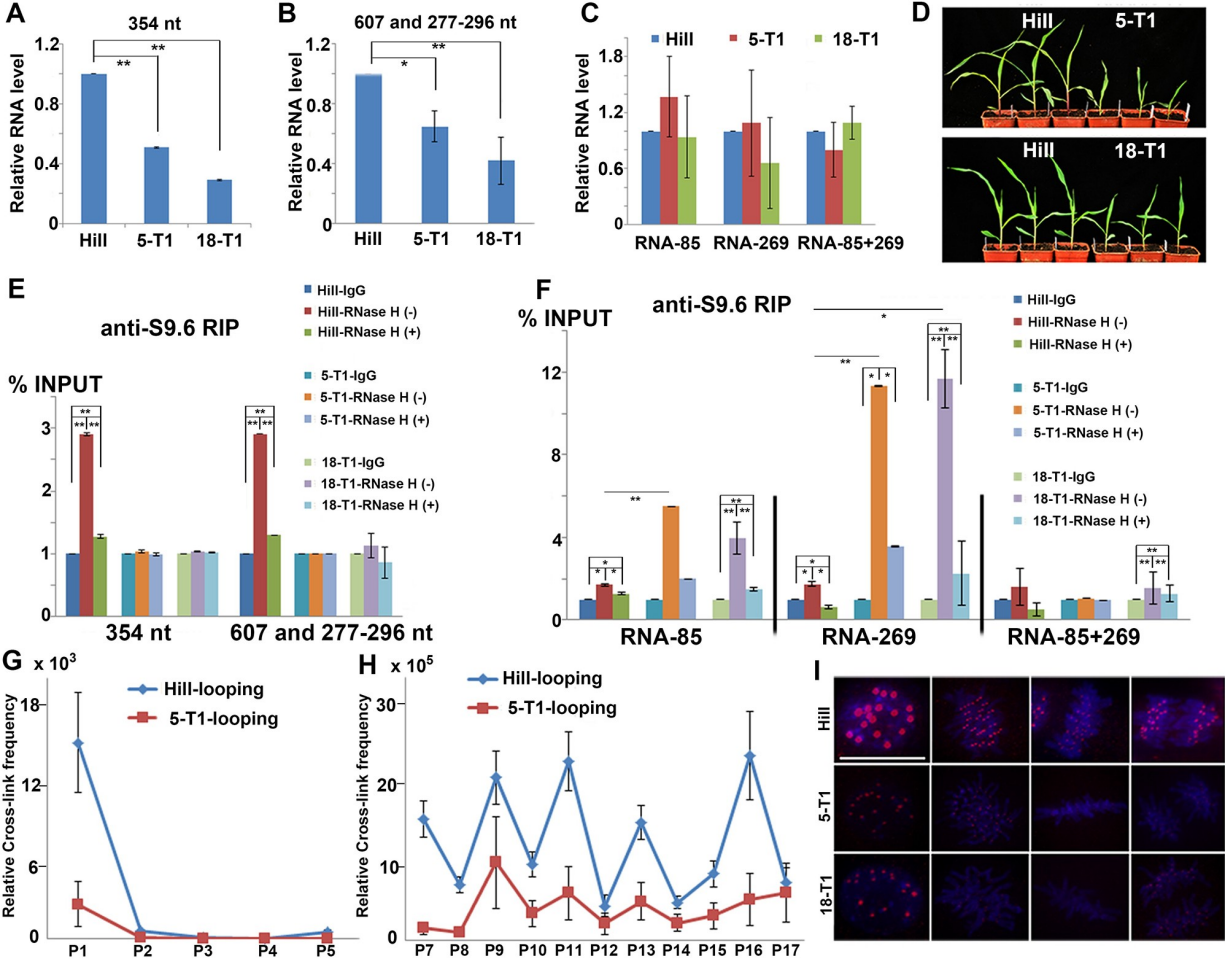
Decreased chromatin loops and CENH3 localizations in the CRM1 regions of the RNAi plants. (A and B) RT-qPCR analysis of the levels of the 354-, 607-, and 277- to 296-nt circular RNAs in the T_1_ generation of RNAi plants 5 and 18, with HiII as the control. (C) Level of linear RNAs (RNA-85, RNA-269, and RNA-85+269) in the T_1_ generation of RNAi plants, with HiII as the control. (D) Seedlings of the T_1_ RNAi plants. (E) Anti-S9.6 RIP quantification of R-loops in the circular CRM1 RNAs in the T_1_ RNAi plants. (F) Anti-S9.6 RIP quantification of R-loops in the linear RNAs in the T_1_ RNAi plants. (G and H) 3C-qPCR analysis of the chromatin interactions inside a single CRM1 element (G) and between two CRM1 elements (H) in the T_1_ generation of RNAi line 5. Data were normalized to the cross-link frequencies of the 3C control template composed of *Dpn*II-digested and ligated centromeric BAC and the internal reference *SAM*. The primers used were the same as in Fig 3. (I) CENH3 signals in the T_1_ RNAi plants. Blue indicates DAPI. Red indicates the CENH3 signals. Bar = 10 μm. In (A–C,) and (E–H), the columns and error bars represent the relative value and standard error of the means (*n* = 3). In (A–C), (E), and (F), *Actin* was used as an internal reference gene, the *P* values were determined using a Student *t* test: *P < 0.05, **P < 0.01. The data underlying this figure can be found in S1 Data. 3C, chromatin conformation capture; BAC, bacterial artificial chromosome; CENH3, centromeric H3 variant; CRM1, centromeric retrotransposon; IgG, Immunoglobulin G; RIP, RNA immunoprecipitation; RNAi, RNA interference; RT-qPCR, reverse transcription quantitative PCR.

To further confirm the potential function of the circular CRM1 RNAs, transgenic RNAi plants were generated via an *Agrobacterium tumefaciens*–mediated transformation. The RNAi target sequence was designed to cover the back-splicing site of the circular RNAs, including a 204-bp sequence of the 269-bp region and the 85-bp sequence (a total of 289 bp). Two positive transgenic events were obtained (RNAi 5 and 18). The levels of the 354-, 607-, and 277- to 296-nt circular RNAs were dramatically decreased in the T_1_ generation of the RNAi plants compared to the wild-type HiII plants (used for the transgenic transformation) (Fig 4A and 4B). The levels of related linear RNAs, including RNA-85, RNA-269, and RNA-85+269, showed no significant changes in the T_1_ RNAi plants (Fig 4C). The undetected changes of the linear RNAs may be caused by weak RNAi effects for repeat sequences with multiple copies, which provided an opportunity to study the roles of circular RNAs. The seedlings of the T_1_ RNAi plants were smaller than those of the wild type and grew normally but slowly during the early stages of growth (Fig 4D).

The levels of chromatin-binding circular CRM1 RNAs were also significantly reduced in the T_1_ generation of RNAi plants 5 and 18 compared to those of the HiII plants (S4A Fig). Correspondingly, the R-loop levels of the 354-, 607-, and 277- to 296-nt circular CRM1 RNAs were dramatically reduced in the RNAi plants, as determined using anti-S9.6 RIP (Fig 4E); however, the R-loop levels of the related linear RNAs (RNA-85 and RNA-269) were increased in the RNAi plants (Fig 4F). The R-loop levels of RNA-85+269 showed no obvious differences between the RNAi plants and the HiII plants (Fig 4F). We checked the ssDNA level in the R-loop sites to determine whether the total R-loop levels in the CRM1 regions were greater in the RNAi plants. In the T_1_ generation of the RNAi plants, the 85- and 64-bp regions inside the 269-bp region were more sensitive to S1 nuclease treatment than those of the HiII plants, suggesting that the ssDNA levels in these two regions were increased (S4B and S4C Fig). The R-loop sites of RNA-85 were located at the 85-bp region, and the R-loop site of RNA-269 was located at the 64-bp region in the RNAi plants. These results revealed that the R-loop sites of circular and linear CRM1 RNAs were similar (Figs 3C and S4C); thus, R-loop formation by these 2 kinds of RNAs was competitive. While the R-loop level of the circular RNA was reduced, the R-loop level of the linear RNAs was increased. The increased ssDNA levels in the RNAi plants indicated that the increased R-loops of the linear RNAs compensated for the decreased R-loops of the circular RNAs, resulting in higher R-loop levels in the CRM1 regions of the RNAi plants.

We further checked for changes in the chromatin loops at the CRM1 regions of the T_1_ RNAi plants. We found that the chromatin-interaction frequencies in the CRM1 regions were decreased in the T_1_ generation of RNAi line 5 compared to those of the HiII plants, while the patterns of chromatin interactions were similar between the two (Fig 4G and 4H). The reduced cross-linking frequencies were obvious between the 2 CRM1 elements in the T_1_ RNAi plants (Fig 4H). These results revealed that the decreased R-loops of the circular CRM1 RNAs led to reduced chromatin loops in the CRM1 regions of the RNAi plants. The increased R-loops of the linear RNAs with one R-loop site could not promote the formation of chromatin loops in the CRM1 regions; thus, the formation of chromatin loops was mainly induced by circular CRM1 RNAs (Fig 3D and 3E).

Throughout the cell cycle, the fluorescence intensity of CENH3 signals in the centromeric regions was reduced in the T_1_ generation of RNAi plants 5 and 18 compared to the HiII plants (Figs 4I, S4D and S4E). In the RNAi plants, the chromosome behaviors and centromere function appeared normal during mitotic nuclear division (Fig 4I). Accurate chromosomal segregation during the cell cycle—maybe due to the reduced CENH3 levels—did not reach the threshold required to disturb centromere behavior. Reduced CENH3 levels in centromeric regions can maintain normal mitosis in plants [58,59]. CENH3 levels in the 269- and 253-bp regions and the nearby sequences were consistently reduced, according to the anti-CENH3 ChIP-qPCR results in the T_1_ RNAi plants (S4F Fig). The 269-bp region contains the R-loop formation sites of the linear CRM1 RNAs. The increased level of R-loops may have decreased CENH3 localization at the same sites in the RNAi plants, which could explain the low ratio of back-spliced CRM1 reads in the anti-CENH3 RIP-seq data of B73 (Table 1). The 253-bp region has no R-loop sites, but it still showed reduced levels of CENH3 association in the RNAi plants. As such, we concluded that the reduced R-loops of the circular CRM1 RNAs led to the increased chromatin binding of linear CRM1 RNAs and decreased chromatin loops in the CRM1 regions of the RNAi plants, which resulted in reduced CENH3 localization.

The T_1_ RNAi plants grew normally at later developmental stages, and their seed setting rate was normal. The seeds had no defects in germination and growth; however, the RNAi plants returned to the wild-type phonotype in the T_2_ generation (S4G Fig). The RNAi vector was still detectable in the T_2_ generation using FISH (S4H Fig), yet the RNA levels of the circular CRM1 RNAs in the T_2_ RNAi plants were similar to those of the HiII plants (S4I Fig). This may be because the RNAi vector was gradually silenced during the development of the T_1_ RNAi plants, meaning that only the T_1_ generation had an obvious phenotype in the early stages of development. The detailed mechanism for this was not analyzed in this work.

### Conserved back-splicing process of retrotransposons in numerous crops

**Fig 5:**
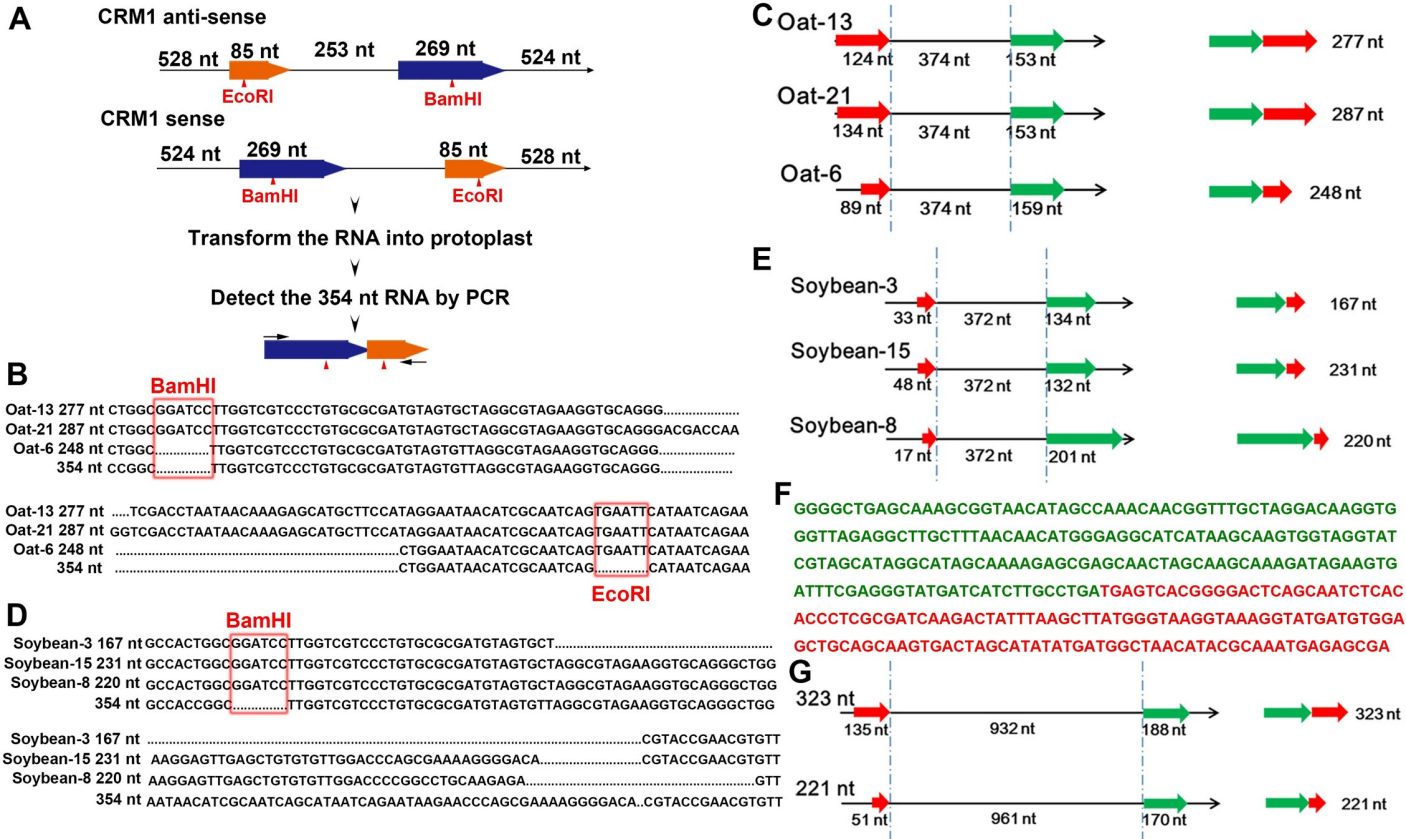
Conserved back-splicing process of retrotransposons in numerous crops. (A) The procedure of protoplast transformation using in vitro–transcribed CRM1 RNA. The digestion sites are marked with red triangles. (B and D) Three 354-nt–like back-spliced RNAs from transformed oat (B) and soybean (D) protoplasts. The red frames mark the labeled digestion sites. The first 3 tracks show the sequences from transformed protoplasts, and the last tracks show the 354-nt RNA sequence. (C and E) The detailed information of the 3 back-spliced sequences from oat (C) and soybean (E) protoplasts. The left panels show the sequence position on the 1,671-nt sequence. The right panel shows the final back-spliced sequences. The dotted lines mark the intermediate region. The green bars show the downstream parts of the 1,671-nt sequence, and the red bars indicate the upstream parts. (F) A 323-nt back-spliced RNA from wheat retrotransposons, consisting of a 288-nt (green) and a 135-nt (red) sequence. (G) Detailed information for the 323-nt and 221-nt back-spliced sequences. The left panel shows the position of the sequence on the retrotransposon, while the right panel shows the final back-spliced sequences. The dotted lines indicate the intermediate region, green bars represent the downstream regions, and red bars indicate the upstream regions. CRM, centromeric retrotransposon; nt, nucleotides.

To assess whether the back-splicing process of retrotransposons is conserved in different plant species, we performed two experiments. First, in vitro–transcribed CRM1 RNA was transformed into the protoplasts of other plant species. A 1,659-bp sequence from the CRM1 DNA of B73, containing a *Bam*HI digestion site in the 269-bp region and an *Eco*RI digestion site in the 85-bp region, was used as the in vitro transcription template, resulting in a total length of 1,671 bp (Fig 5A). The in vitro–transcribed 1,671-nt CRM1 RNAs from both the sense and antisense strands were used for the transformation, and then the expected back-spliced products with labeled digestion sites were checked after protoplast transformation (Fig 5A).

Oat (*Avena sativa*) was selected for the protoplast preparation due to its ability to stably transmit maize chromosomes [60,61]. Similar back-spliced 354-nt RNA with labeled sites was detected in the protoplasts after transformation with the sense strand 1,671-nt RNA (S5A Fig). Differently sized back-spliced sequences with various back-splicing sites were also identified in the oat protoplasts (Fig 5B and 5C). Monocotyledonous crops such as rice (*O*. *sativa*), common wheat (*Triticum aestivum*), and sorghum (*Sorghum bicolor*) were also used for protoplast transformations. Alternative back splicing of the CRM1 RNA occurred in the samples transcribing the sense strand of all 3 species (S5B–S5G Fig). A similar back-splicing process was confirmed even in dicotyledonous crops, such as soybean (*Glycine max*) (Figs 5D, 5E and S5H).

We next checked the endogenous back-spliced RNAs from retrotransposons according to the RNA sequencing (RNA-seq) data. A 323-nt endogenous RNA showing a back-splicing pattern was identified from the wheat retrotransposon RLG_Sumaya_116F2-1, based on the lncRNA sequencing (lncRNA-seq) data of *T*. *urartu* (Fig 5F). The 323-nt RNA consisted of a 188-nt sequence and a 135-nt sequence (Fig 5F and 5G). An alternative 221-nt splicing product was also detected in the total RNA of common wheat (Fig 5G). The distributions of the 188- and 135-bp sequences differed between in the AA, BB, and DD subgenomes of common wheat. The AA subgenome had the highest copy number of these sequences, whereas the BB subgenome had the lowest copy number (S5I Fig). These results clearly demonstrated that the alternative back-splicing process in retrotransposons may be conserved in numerous crops.

## Discussion

**Fig 6:**
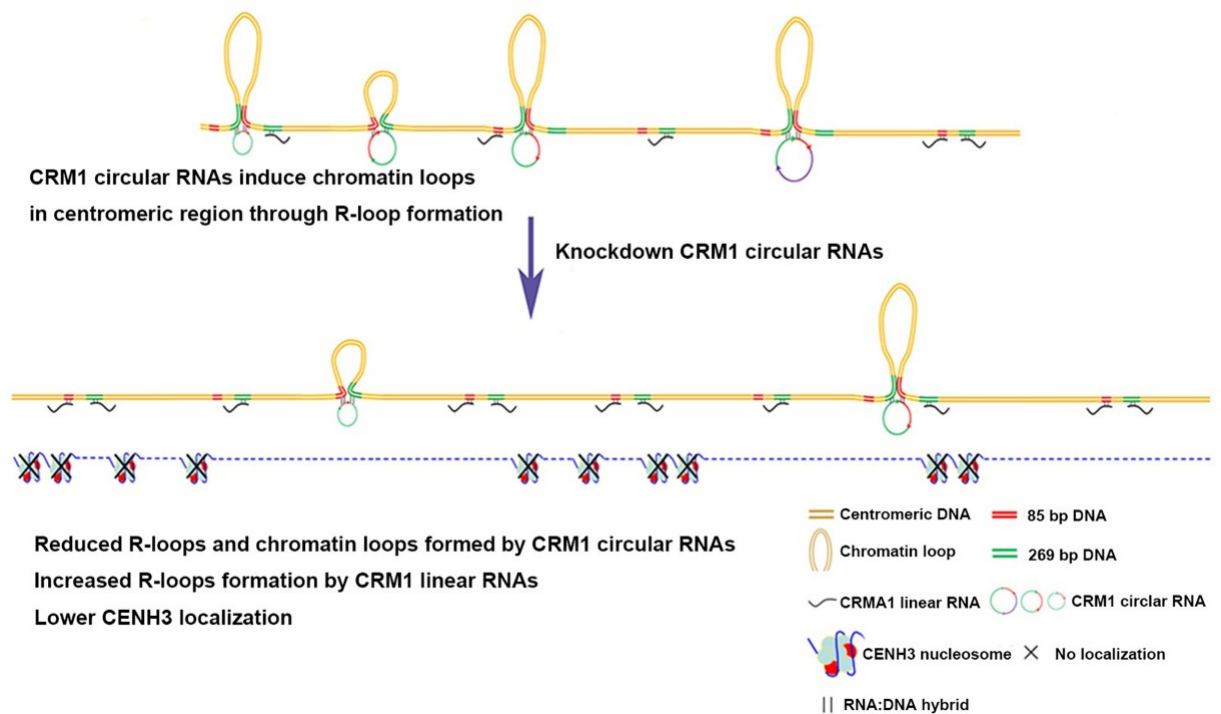
The model for roles of circular CRM1 RNAs in centromere structure and function. Circular CRM1 RNAs can bind to the chromatin through R-loops to induce chromatin loop formation. The chromatin binding of circular RNAs can repress the formation of R-loops by related linear RNAs. Higher numbers of R-loops and lower numbers of chromatin loops lead to lower levels of CENH3 localization in the centromere. CENH3, centromeric H3 variant; CRM, centromeric retrotransposon.

During maize evolution, specific retrotransposons were inserted and rearranged in the centromeric regions [62]. Many studies have shown the predominant function of CRM2 in maize [8,11,13,14,37], but we wondered why so many copies of CRM1 remained active during maize centromere evolution [11]. The results of this work show that multiple RNAs from CentC and CRM elements bind to maize centromeres. In addition to large numbers of linear RNAs, we also identified circular RNAs produced from the CRM1 elements. No circular RNAs have previously been reported to arise from CRMs. These circular CRM1 RNAs bind to maize centromeres through R-loops to promote the formation of chromatin loops (Fig 6). Higher numbers of R-loops and lower amounts of chromatin loops were shown to result in decreased CENH3 localization (Fig 6). Our work reveals the potential function of CRM1 in centromere structure and function and indicates that CRM1 may have helped to maintain a stable chromatin environment during centromere evolution. Different repeat sequences in the centromeric regions may have different functions, such as providing sites for CENH3 localization, inducing proper chromatin structure, triggering chromatin transcription, and so on. All the potential RNA, DNA, nucleosomes, and chromatin-binding factors may work together to maintain centromere function, and these factors may be coevolved.

Many centromeric RNAs have been detected in different species [17,29–37]. In human centromeres, the transcription of the centromeric α-satellite occurs at late mitosis into early Gap 1 (G1) phase, which coincides with the deposition of new CENP-A [32]. In budding yeast, the peak expression level of centromeric RNA occurs during Synthesis (S) phase, which is concurrent with CENH3^Cse4^ (Chromosome segregation protein 4) deposition [35,63]. In *Arabidopsis*, CENH3 deposition occurs at the G2 phase [64]; however, until now no study has accurately documented the time of CENH3 deposition in maize. According to the published work, circular RNA expression is independent of their parental transcripts [65]. In our work, the time of circular RNAs transcription and CENH3 localization in centromeres may be analyzed in the future.

The level of back-spliced CRM RNA is low in the anti-CENH3 RIP sample. In most cases, circular RNAs are transcribed at low levels compared to linear transcripts [65]. One recent work has studied the full length of circular RNAs and their expression networks using 132 RNA-seq libraries; this work revealed that a large amount of sequenced data are needed for the accurate measurement of the relative expression between circular and linear RNA [65]. However, the back-splicing process of retrotransposons is not clear in plants, and we cannot detect back-spliced reads from CRM elements in the RNA-seq data that were enriched for circular RNA with RNase R treatment. We cannot obtain the accurate ratio of circular/linear RNAs from CRMs at present. Recent work in budding yeast showed that RNA from cen8 has a copy number of 0.002 molecules per cell, and the misexpression of this RNA can lead to chromosome loss [35]. This work indicated that the centromeric RNAs could have important functions even though they are not abundant. In our research, the circular CRM1 RNAs exist in cells with quite low copies, and they perform functions in centromeric chromatin.

In maize, we observed the formation of the R-loops by CRM1 RNA and their regulation in the centromere. Studies of yeast demonstrated that R-loops are linked to H3-Ser10 phosphorylation and chromatin condensation, especially at centromeric and pericentromeric regions [41]. The chromatin structure and chromatin state can also affect R-loop formation in yeast [42]. Our results showed that both circular and linear RNA from the CRM1 elements form R-loops in centromeric regions, revealing that R-loops are components of centromeric chromatin. Our work also revealed that the circular CRM1 RNAs and the related linear RNAs compete to form R-loops in the centromeric regions (Fig 6). As the circular and linear RNAs may be transcribed at different times [65], the formation of R-loops by these 2 kinds of RNA may be temporally regulated. The abundance of R-loops in the centromere may also be dynamically regulated by the RNA level. R-loops can induce changes in DNA methylation at promoters, thereby regulating gene transcription [44]. Therefore, apart from well-known chromatin regulators, such as histone modifiers, chromatin assembly factors, and RNAPII [26,27,66], R-loops may also affect the chromatin modification and transcription process in centromeric chromatin. Proper chromatin modification and transcription states are important for the deposition and maintenance of CENH3 [66]. Histones H3.1 and H3.3 coexist with CENH3 in the centromeric regions during interphase, the localization of which is precisely regulated by their chaperones [66]. Despite this insight, the mechanisms by which the chaperones recognize the correct sites for histone deposition have not yet been fully elucidated [67]. R-loops in the centromeric regions may provide markers for histone deposition.

Chromatin loops can be induced in the centromeric regions by circular CRM1 RNAs (Fig 6). The chromatin-interaction frequencies between two CRM1 elements (which can be more than several kilobase) were greater than within a single CRM1 element (which is less than 600 bp), suggesting that large chromatin loops are more common in the CRM1 regions. Chromatin loops can have a widespread impact on transcription [68]. It is suggested that chromatin loops in the centromere may influence transcription and the binding of chromatin-regulating factors, which may affect CENH3 localization. Furthermore, the CENP-A chaperone interacts with the condensing II complex during G1 phase to promote CENP-A deposition [69]. The chromatin loops are therefore involved in the organization of centromeric chromatin. Work in animals has shown that the zinc finger protein CCCTC-binding factor (CTCF) is involved in mediating chromatin interactions and that cohesins stabilize chromatin loops [70]. Cohesin- or condensin-dependent chromosome interactions are organized during cell division [71]. No proteins similar to CTCF have been found in plants yet. In this work, the R-loops of circular RNA were found to induce the formation of chromatin loops, unveiling a new feature of centromeric chromosomal organization. The chromatin loops and R-loops in the CRM1 regions may function together to provide a proper centromeric chromatin environment for CENH3 localization, which may be the reason for the insertion of CRM1 elements during evolution.

CRM1 is extensively distributed along the centromere, as are the R-loops and chromatin loops associated with the CRM1 elements. Each centromere with a specific CRM1 composition should have its own distinguishing centromere architecture and CENH3 localization patterns, which may be important for centromere pairing during the early meiotic stage [72] and dynamic centromere behavior during the cell cycle [73,74]. When hybridization between 2 different genomes occurs, changes in centromere transcripts may lead to changes in the centromeric chromatin structure and finally in centromere function. During artificial chromosome design, the composition and arrangement of centromeric sequences may play roles not only in supporting the chromatin skeletons for CENH3 deposition but also in sustaining a stable structure for CENH3 maintenance. Our results therefore connect centromere structure and function based on CRM elements.

During de novo centromere formation, CENH3 may initially be deposited in many positions [19–21]. According to our current results, stable de novo centromeres should be formed at positions with appropriate chromatin structures and states to ensure the stable localization of CENH3 during cell division. If the centromeric chromatin structure and state are not optimal, CENH3 will not be deposited at regular intervals, and the corresponding chromatin fragment will be lost during later cell cycles. The inactive centromeric region may be regulated by aberrant levels of R-loops and chromatin loops, together with some chromatin modifications [75], thereby blocking CENH3 localization.

In the centromeric region, abundant RNAs from the centromeric CRM elements were identified. Because the repeat sequences are present in many copies, especially centromeric repeats, the dedicated tools for the detection of circular RNA from the next-generation sequencing data are not suitable for analyzing circular RNA originating from repeats [76]. Instead, we identified back-spliced reads using BLAST searches of the next-generation sequencing data. Only back-spliced centromeric RNAs were studied in this work; however, RNA from both the LTR regions and the gene body regions of the CRM elements may also have potential functions in centromeric structure and function. Retrotransposons are a major component of plant genomes, contributing to genome stability and adaption during evolution [77,78]. Our results showed that the process of back splicing in retrotransposons has been conserved in numerous crops over the course of evolution. Elucidating the interactions between retrotransposon RNA and chromatin organization may therefore promote a greater understanding of the function of retrotransposons in genome evolution and stability.

## Materials and methods

### Plant materials

The inbred line B73 was used for all the analyses performed in maize. The first generation of the hybrid HiII A × B was used for the RNAi transgene transformation. The rice restorer line Minghui63, sorghum inbred line BTx623, common wheat cultivar Chinese Spring, oat cultivar Baiyin11, and soybean cultivar Williams82 were used for the protoplast transformations.

### Anti-CENH3 ChIP-seq and qPCR analysis

This experiment was conducted as previously described [79]. About 10 g of young B73 leaves was used for the ChIP assays. The enriched DNA samples were sequenced using the Illumina Hiseq 2000 platform. Approximately 30 to 40 million 101-bp paired-end reads were mapped to the maize B73 RefGen_v4 using BWA software with default parameters as previously described [80]. The uniquely mapped reads with MAPQ values larger than 20 were used for further analysis. The distributions of ChIP-seq reads were calculated using the reads per million (RPM) value and were displayed with Integrative Genome Viewer (IGV). For anti-CENH3 ChIP-qPCR, samples collected throughout the procedures with IgG binding were used as negative controls. Actin was used as an internal reference gene for normalization. The primers are listed in S2 Table. The paired-end reads from anti-CENH3 ChIP and input data were joined with SeqPrep (https://github.com/jstjohn/SeqPrep) with the parameter “-q 30 -L 25,” and the merged reads with remaining unmerged paired-end reads underwent BLAST to the assumed 354-bp sequence using the parameter “-e value 1 × 10^−5^.” The positions of matched reads were compared to the location of the junction site to detect whether there are reads covering the back-splicing site. The total centromere sizes in maize were estimated with the CENH3-binding regions using the ChIP-seq data; centromere coverage was determined according to the total number of sequencing reads divided by the centromere sizes.

The paired-end reads generated from the anti-CENH3 ChIP-seq dataset are available under the Gene Expression Omnibus (GEO) database (GSE124242). The public anti-CENH3 ChIP-seq datasets were obtained from GEO database with accession numbers SRR3018834, SRR2000635, and SRR2000640, and the input data were obtained with accession number SRR2000648. The public raw reads of Pacbio and Illumina whole-genome sequencing datasets were from SRX1472849 and SRX1452310.

### RIP and RIP-seq data analysis

Young leaves were cross-linked in 0.4 M sucrose, 10 mM Tris-HCl (pH 8), 1 mM EDTA, 1 mM PMSF, and 1% formaldehyde in a vacuum for 30 min, after which the reaction was terminated by adding 2 M glycine. The leaves were washed 3 times with RNase-free water, and Kimwipes (Kimberly-Clark Professional) were used to remove water drops from the leaves. The leaves were then transferred to liquid nitrogen and ground into a fine powder. The following experiment was carried out using the native ChIP protocol as previously described with slight modification [79]. The chromatin was digested into single nucleosomes using 0.02 U/μl DNase I (RNase-free) at 37°C. Dynabeads Protein A (Thermo Fisher, Category Number 10001D) was used for binding the anti-CENH3 antibody. After washing the beads incubated with the antibody and chromatin fragments, the beads were eluted twice at 65°C for 15 min using elution buffer containing 50 mM Tris-HCl (pH 7.5), 10 mM EDTA, 200 mM NaCl, and 1% SDS. The eluted fractions were combined, and Proteinase K (Invitrogen, Category Number 25530–49) was added to relieve cross-linking at 37°C overnight, after which the RNA was extracted using TRIzol reagent, and the residual DNA was removed using RNase-free DNase I (NEB, Category Number M0303S).

For the clone selection, the RNA was reverse transcribed with Superscript III (Invitrogen, Category Number 18080–044), and first-strand cDNA was generated using random primers. The PCR products were ligated to the T-vector using a pEASY-T1 Simple Cloning Kit (Trasngen, Category Number CT111-02). The primers are listed in S2 Table. For the RIP-seq, rRNA was removed using the Ribo-Zero rRNA Removal Kit (Illumina, Category Number MRZ11124C). The cDNA libraries of RIP samples were prepared using the standard manual provided in the NEB Next Ultra RNA Library Prep Kit for Illumina (NEB, Category Number E7530S). The samples were applied to the Illumina HiSeq 2500 sequencing system, generating approximately 37 million and 20 million 125-bp paired-end reads for RIP and input samples, respectively.

The methods to join the paired-end reads from anti-CENH3 RIP and input data and BLAST to different sequences were the same for the ChIP-seq reads as mentioned earlier. The number of mapped reads for each unit of the different repeat sequences was counted, and the relative enrichments of each sequence in RIP samples were calculated using the ratios normalized by input reads. The paired-end reads from the anti-CENH3 RIP-seq dataset have been made available, and the input dataset was available in the GEO dataset (GSE137701).

The same strategy was applied to discover the back-spliced RNAs from RNA-seq datasets of wheat. Wheat retrotransposons were identified using LTR_STRUC software. The method used to identify the novel back-spliced retrotransposon RNAs from wheat was performed as described previously. The paired-end reads used for wheat were obtained from the lncRNA-seq dataset of *T*. *urartu* (GSE137701).

### DNA and RNA extraction

The genomic DNA of B73 was extracted using hexadecyltrimethyl ammonium bromide (CTAB). Nuclear DNA was obtained from unbroken nuclei extracted using the 3C method [81]. The nuclei were treated with Proteinase K (Invitrogen, Category Number 25530–49) in elution buffer (50 mM Tris-HCl [pH 7.5], 10 mM EDTA, 200 mM NaCl, and 1% SDS) overnight at 37°C and then extracted using a phenol-chloroform extraction approach.

Total RNA was extracted using TRIzol reagent, and the first-strand cDNA was produced using MMLV reverse transcriptase (Promega, Category Number M170A). The RIP-RNA and protoplast-transformed RNA was extracted using TRIzol reagent and reverse transcribed using Superscript III reverse transcriptase. Chromatin RNA was extracted using the same method applied for ChIP. The chromatin was treated with Proteinase K in RNA elution buffer (50 mM Tris-HCl [pH 7.5], 10 mM EDTA, 200 mM NaCl, and 1% SDS) overnight at 37°C and then extracted using TRIzol reagent.

### RNase R treatment

For the detection of circular RNA, the total RNA was treated to remove mRNA and rRNA, as previously described [49]. A total of 20 μg total RNA was extracted using TRIzol reagent, and then the mRNA was removed using the Poly(A) mRNA Magnetic Isolation Module (NEB, Category Number E7490S). rRNA was subsequently removed using a Ribo-Zero rRNA Removal Core Kit and Ribo-Zero rRNA Removal Reagent (plant leaf) (Illumina, Category Number RZPL1224). The residual RNA was treated with 5 U of RNase R (Epicentre, Category Number RNR07250) at 37°C for 3 h. The RNA was then extracted using TRIzol reagent and reverse transcribed with Superscript III. The primers used for RT-PCR are listed in S1 and S2 Tables.

### RNA purification using biotinylated antisense oligos

The experimental procedures for RNA antisense purification followed the protocols developed by Jesse Engreitz (https://www.guttmanlab.caltech.edu/protocols-RAP.php) and other previously published methods [82]. Both sense and antisense oligos were used to capture complementary RNAs from the total RNA of B73 leaves. The probes were directly synthesized by Invitrogen, with biotinylated dNTPs added to the 3′ end of the oligos. Four biotinylated probes from the 354-nt sequence were used together to capture the complementary RNAs. These probes corresponded to the 1- to 60-bp, 121- to 180-bp, 244- to 294-bp, and 301- to 356-bp regions of the 356-bp DNA (S4 Table). RNAs captured using both the biotinylated sense and antisense oligos were mixed together for PCR detection. The ssDNA probes are listed in S4 Table. The primers used to detect the full length of the 354-nt RNA from purified RNAs are listed in S3 Table. The 354-nt circular CRM1 RNA was submitted to NCBI GenBank with the accession number MN481933.

### Northern blotting

The RNA used for northern blotting was prepared following the purification of the antisense probe. The Dig High Prime DNA Labeling and Detection Stater Kit I (Roche, Category Number 1745832910; for color detection with NBT/BCIP) was used for signal detection in northern blotting, following the protocol supplied with the kit. Digoxin-labeled sense probes were used in northern blotting to detect the RNA purified by the biotinylated sense oligos. The digoxin-labeled antisense probes were used in northern blotting to detect the RNA purified using the biotinylated antisense oligos. The RNA was run on a 3% denaturing formaldehyde agarose gel. The probes are listed in S5 Table.

### RNase H treatment

To confirm that circular RNAs can form RNA:DNA hybrids, 4 μg of chromatin-binding RNAs was treated with 120 U of RNase H (Takara, Category Number 2151) at 37°C for 3 h. The RNA was then purified using a phenol-chloroform extraction, and Superscript III reverse transcriptase was used for reverse transcription. For the RT-qPCR, RNA not treated with RNase H was used as a control, and *Actin* was used as an internal reference gene for normalization. The primers used are listed in S2 and S3 Tables.

### RIP using the S9.6 antibody

Chromatin RNA was extracted according to the method described earlier. The dsRNA was removed using RNase III (Thermo Fisher Scientific, Category Number AM2290). A total of 5 μg RNA with or without RNase H treatment was used for each immunoprecipitation (IP) experiment, and another 5 μg RNA without antibody binding was used as the control. A 3-μl aliquot of RNase H was added to the RNase H–treated sample, and 3 μl DEPC-treated H_2_O was added to the samples that did not receive RNase H or the antibody treatment. The RNA was then precleaned using Protein A/G Plus Agarose (Santa Cruz Biotechnology, Category Number sc-2003) in IP buffer (10 mM sodium phosphate buffer [pH 7], 140 mM NaCl, and 0.1% Tween 20) for 4 h, prior to the addition of 4 μg S9.6 antibody (Kerafast, Category Number ENH001) and an overnight incubation. A 100-μl aliquot of Protein A/G Plus Agarose was used to purify the antibody–RNA complex during 2-h incubation. After washing the Protein A/G Plus Agarose 3 times with IP buffer, the bound RNA was eluted twice using RNA elution buffer (50 mM Tris-HCl [pH 7.5], 10 mM EDTA, 200 mM NaCl, and 1% SDS) at 65°C for 15 min. The RNA was extracted in a phenol-chloroform extraction and reverse transcribed using Superscript III. The primers used for qPCR are listed in S2 and S3 Tables.

### S1 nuclease treatment

Nuclear DNA was extracted as described earlier. A total of 5 μg of nuclear DNA was treated with 0, 1,000, and 2,000 U of S1 nuclease (Invitrogen, Category Number 18001016) for 3 h at 37°C. The samples were extracted using a phenol-chloroform extraction. The qPCR primers used to detect the ssDNA region are listed in S6 Table.

### T7 endonuclease I treatment

Nuclear DNA was extracted as described earlier. A total of 5 μg of nuclear DNA was treated with 100 U of T7 endonuclease I overnight at 37°C. The DNA polymerase I large (Klenow) fragment (NEB, Category Number M0210) was added to fill in the ends and incubated at 25°C for 15 min. After that, the enzyme was inactivated at 75°C for 20 min. T4 DNA ligase (NEB, Category Number M0202) was supplemented to ligate the DNA fragments at 16°C overnight, which could then be used as a PCR template to detect the shorter sequence generated by T7 endonuclease I (NEB, Category Number M0302) cleavage. The primers are listed in S7 Table.

### AFM observation

Circular CRM1 RNAs were prepared by purification from the total RNAs using biotinylated sense oligos targeting the back-splicing site and were treated with 5 U of RNase R at 37°C for 2 h. For AFM preparation, all samples were performed in a solution of 10 mM Tris-HCl (pH 7.5) with the RNA concentration of 3 ng/μl. A 20 μl sample containing 5 mM MgCl_2_ was incubated on the surface of freshly cleaved mica for 5 min, rinsed with 200 μl of Milli-Q filtered ultrapure water, and dried with a gentle stream of nitrogen gas. All images were obtained under ambient air conditions using a Bruker MultiMode 8 AFM with a nanoscope IIIa controller in ScanAsyst mode. The heights, widths, and circumferences of these circular RNAs were calculated with this custom software. Estimating lengths of circular RNAs from the AFM images was performed according to a previously described method [83].

### 3C in maize

The 3C sample was produced according to a previously described method [80], and the DNA was digested with the enzyme *DpnII* (NEB, Category Number R0543). The quantity and quality of the DNA samples were normalized to the internal reference gene *SAM* [56]. The 3C control template includes the *DpnII-*digested and ligated centromeric BAC (ZMMBBb0497C16) DNA containing CRM1 and the PCR products of the *SAM* locus amplified from the 3C DNA sample [57]. The no-looping control was designed using nuclear DNA [84]. Primers used for 3C-PCR and 3C-qPCR are listed in S8 and S9 Tables.

### Generation of transgenic RNAi lines

The RNAi vector was generated by adding a 289-bp sequence containing the back-splicing site to the pUC-RNAi vector [85], using *XhoI* (NEB, Category Number R0146) and *BglII* (NEB, Category Number R0144) as well as *BamHI* (NEB, Category Number R0136) and *SalI* (NEB, Category Number R0138) digestion. The constructed sequence was then transferred into a pCambia3301 vector via a *PstI* (NEB, Category Number R0140) digestion for the *Agrobacterium*-mediated transformation of maize. The primers are listed in S10 Table. The transformation of young embryos and the selection of transgenic plants were performed according to a previously described procedure [86]. The transgenic RNAi plants were identified via FISH using a probe for the pCambia3301 vector and an RT-PCR with the primers listed in S1 and S3 Tables.

### In vitro transcription

The 1,671-bp CRM1 DNA sequence amplified from maize genomic DNA was cloned into the pET-30a vector using *XbaI* (NEB, Category Number R0145S) and *HindIII* (NEB, Category Number R3104S) digestion. A *BamHI* digestion site was added to the 269-bp region, and an *EcoRI* digestion site was added to the 85-bp region. The plasmid was then linearized using *XhoI* for in vitro transcription. In vitro transcription was performed according to the protocol given in the manual for T7 RNA polymerase (NEB, Category Number M0251). The primers are listed in S11 Table.

### Protoplast transformation

Maize protoplast transformation was performed as previously described [85]. A total of 5 μg of in vitro–transcribed RNA was used for each transformation. The method used for soybean, rice, and sorghum was the same as that used for maize. For wheat and oat, the young leaves were cut into slices and soaked in 0.6 M mannitol for 10 min before the addition of the enzyme solution. After cultivation for more than 8 h, the total RNA was extracted using TRIzol reagent.

### FISH and immunostaining assays

FISH and immunostaining assays were performed as previously described [18]. The 354-bp sequence was labeled with Alexa Fluor-488-5-dUTP (Thermo Fisher Scientific, Category Number C11397) using a nick translation to detect its location in the centromeric regions. The pCambia3301 vector was also labeled with Alexa Fluor-488-5-dUTP in a nick translation to identify the transgenic RNAi plants. Maize anti-CENH3 antibodies were used as previously described [21]. Immunostaining images were taken as a confocal z-stack (Zeiss Cell Observer SD) and processed using Adobe Photoshop CS 6.0.

Thirty cells from interphase and 15 cells from mitosis were examined for each transgenic line to identify the centromere fluorescence intensity of the CENH3 signals with ImageJ software [87]. Significant differences were calculated with a two-tailed Student *t* test.

## Supporting information

S1 Fig Back-spliced RNA from CRM1 in the centromere. (A) The strategy of joining the paired-end reads for BLAST searching the centromere retrotransposons. (B) Relative enrichment of reads from the anti-CENH3 RIP-seq data associated with CRM elements, CentC repeats, 2 expressed centromeric genes (Zm00001d030471 and Zm00001d004256), and the 2 unexpressed genes (Zm00001d004248 and Zm00001d030471) located in centromere regions near CRM1 elements. The dotted line represents the value of no enrichment. (C) DNA-FISH of the 354-bp clone sequence. Blue indicates DAPI. Green indicates the sequence. Bar = 10 μm. (D) RT-PCR analysis of the 354-nt RNA without and with reverse transcription. (E and F) The distribution of the 354-bp sequence on Chromosome 2 (panel E) and Chromosome 5 (panel F). The *x* axis in the first panel represents the positions along Chromosome 2 (panel E) and Chromosome 5 (panel F). The *x* axis in the second panel represents the enlarged view of cen2 (panel E) and cen5 (panel F) as illustrated by CENH3 enrichment. The first track of each panel represents the centromeric region. The other 4 tracks represent the distributions of the 354-bp, CRM1, CRM2, and CentC, respectively. The red box indicates the centromeric region. The peak heights in each track represent the RPM value (0–1). (G) Anti-CENH3 ChIP-qPCR shows the enrichment of the 269-bp and 253-bp DNA in CENH3 binding regions. “right-300 bp” represents the 300-bp DNA on the right of the 607-bp sequence. *Actin* was used as an internal reference gene. The columns and error bars represent the relative value and standard error of the means (*n* = 3), respectively. *P* values were determined by Student *t* test: *P < 0.05, **P < 0.01. The data underlying this figure can be found in S1 Data, S1 Raw Images, and on Github (https://github.com/sxx-ying/maize-centromere-circRNA).
(TIF)

S2 Fig Full length of circular CRM1 RNAs. (A) The sequences amplified using primers F4+R2. The first 2 lines represent 65-nt sequence from the 269-nt region and the 85-nt sequence, respectively. The third line represents the amplified 97-nt sequence. (B) Divergent primers F3+R3 were used to detect the existence of the 354-nt circular RNA. (C) The sequences amplified using primers F3+R3. In (A) and (C), the upper 2 lines are 65-nt sequence in the 269-nt and 85-nt sequence, respectively. The third line is the amplified sequence. (D) The sequences amplified using primers F2+R2. The first line represents the amplified 590-nt sequence, and the second line represents the 607-nt sequence. (E) Divergent primers F1+R1 were used to detect the existence of the 607-nt circular RNA. (F) The sequences amplified using primers F1+R1. The first line is the amplified sequence and the second line is the 607-nt sequence. (G) The shorter sequences amplified with primers F2+R2. Five sequences are shown. (H) Divergent primers F2+R3 confirmed the existence of the 277- to 296-nt circular RNA. The 17- to 27-nt sequence from the 85 nt was connected to the 5′ end of the 269-nt sequence in the amplified sequences. In (B), (E), and (H), the upper panel represents the positions of primers on the 354-nt sequence, and the lower panel represents the amplified sequences. (I) The sequences amplified with primers F2+R3. Five sequences are shown. The green bars and green lines represent the 269-nt sequence in the 354-nt and the amplified sequence, respectively. The red bars and red lines represent the 85-nt sequence in the 354-nt and the amplified sequence, respectively. The purple lines represent the intermediate 253-nt sequence. (J) Distribution of the heights, widths, and circumferences of the circular RNAs. Each point indicates a circular RNA. About 30 molecules are calculated. Mean and standard error of mean indicated. The data underlying this figure can be found in S1 Data.
(TIF)

S3 Fig Circular CRM1 RNAs induce chromatin loops in the centromere. (A) RNA:DNA hybrids formed by circular CRM1 RNAs were checked by RNase H treatment. Chromatin-binding RNA was used for confirmation. (B) The potential cases for R-loop formation by circular CRM1 RNAs. The blue circles represent circular CRM1 RNAs. (C) Detection of the R-loop structure by T7 endonuclease I digestion and subsequent ligation. The red arrows show the shortened sequences. (D) Shortened sequence (showed by the rectangle with dotted line) obtained after T7 endonuclease I treatment and DNA ligation. The arrows on 2 sides show the primer positions. The detailed sequences of the shorter PCR bands were shown in the lower panel. The upper 3 tracks show the sequences of CRM1, the 85 bp, and the 269 bp, respectively. The fourth track shows the shorter sequence amplified by Primer 1. The last 2 tracks show the shorter sequences amplified by Primer 3. (E) The *DpnII* digestion sites on the 607-bp region and the surrounding regions. (F) RNA-85, RNA-269, and RNA-85+269 were sensitive to RNase R treatment. The right panel shows the positions of the primers. (G) Anti-S9.6 RIP-qPCR was used to confirm the R-loop formation by linear CRM1 RNAs. Chromatin-binding RNA was used for RIP. (H) The percentages of chromatin-binding RNA in the total RNA for the CRM1 RNAs. In (A) and (G), *Actin* was used as an internal reference gene, the columns and the error bars represent the relative value and standard error of the means (*n* = 3). *P* values were determined by Student *t* test: *P < 0.05, **P < 0.01. In (B), (D), (E), and (F), the red, yellow, and green bars represent the 85-bp, 253-bp, and 269-bp region, respectively. The black lines represent the left and right sides of the 607-bp region. The data underlying this figure can be found in S1 Data and S1 Raw Images.
(TIF)

S4 Fig Decreased chromatin interactions and CENH3 localizations in CRM1 regions. (A) RT-qPCR shows that chromatin-binding levels of the 354-nt, 607-nt, and 277- to 296-nt circular RNAs are reduced in the RNAi plants. (B) The regions chosen for detecting the ssDNA sites are marked as 85–1, 253–1, 253–2, 253–3, 269–1, and 269–2. The red bars show the ssDNA regions confirmed in (C). (C) The ssDNA sites confirmed by 1,000 U S1 nuclease treatment. The DNA with no S1 nuclease treatment was used as the control template; 607-left was used as an internal reference gene. (D and E) Quantification of the CENH3 fluorescence intensity in the T_1_ generation of RNAi plants and HiII. Thirty cells from interphase (D) and 15 cells in mitosis (E) were measured to quantify the CENH3 signals. (F) Anti-CENH3 ChIP-qPCR analysis showed reduced CENH3 localization in CRM1 regions in the T_1_ generation of RNAi plants 5. (G) Seedling phenotypes of the T_2_ generation of the RNAi plants. The left 3 seedlings are from HiII and the right 3 seedlings are from the T_2_ generation plants in each panel. (H) FISH detection of the RNAi vector in the T_1_ and T_2_ generation of the RNAi plants. Blue indicates DAPI. Green indicates the vector signals. Bar = 10 μm. (I) The RNA levels of CRM1 circular RNAs in the T_2_ generation of RNAi plants. In (A), (C), (D), (E) and (I), HiII is the control. In (A), (F), and (I), *Actin* was used as an internal reference gene. In (A), (C), (D), (E), (F), and (I), the columns and error bars represent the relative values and standard error of the means, respectively. *P* values were determined by Student *t* test: *P < 0.05, **P < 0.01. The data underlying this figure can be found in S1 Data.
(TIF)

S5 Fig The back-splicing process in retrotransposon is conserved in crops. (A, B, E, and H) The sense-strand–transcribed CRM1 RNA can be spliced into the 354-nt–like back-spliced RNA after being transformed into oat (A), rice (B), wheat and sorghum (E), and soybean (H) protoplasts. (C and F) The 354-nt–like back-spliced RNAs from rice (C) and wheat and sorghum (F) protoplast transformation. The red frames mark the labeled digestion sites. The first track shows the 354-nt RNA and the other 3 tracks show the back-spliced sequences in protoplasts. (D and G) Detailed process of the back splicing in rice (D) as well as wheat and sorghum (G) protoplast. The red arrows show the upstream sequence, and the green arrows show the downstream sequence. The left panel shows the sequence positions on the 1,671-nt sequence, and the right panel shows the final spliced sequences. (I) The distributions of the 323-bp sequence (consisted of 188-bp and 135-bp sequence) in the AA, BB, and DD subgenome of common wheat. The *x* axes in each panel represent positions along Chromosome 1A, 1B, and 1D. The first 3 tracks of each panel represent distributions of the 323-bp, 188-bp, and 135-bp sequence along the whole chromosome. The other 3 tracks represent several detailed positions. The data underlying this figure can be found in S1 Data, GSE137701, and on Github (https://github.com/sxx-ying/maize-centromere-circRNA).
(TIF)

S1 Raw Images Raw annotated immunoblot and electrophoretic gel images for Figs 2A, 2E, 2F, 3F, S1D, S3C, S3F, S5A, S5B, S5E and S5H. (PDF)

S1 Data Numeric data underlying the graphical plots shown in Figs 3A, 3C, 3G, 3H, 4A–4C, 4E–4H, S1B, S1G, S2J, S3A, S3G, S3H, S4A–S4F, and S4I. (XLSX)

S1 Table Primers used for PCR detection. (DOCX)

S2 Table Primers used for ChIP-qPCR and RT-qPCR. (DOCX)

S3 Table Primers used for the full-length detection of 354-nt RNA. (DOCX)

S4 Table Probes for RNA purification (with biotin labeled on the 3′ end of the probes). (DOCX)

S5 Table Probes for northern blotting (with digoxin labeled on the 3′ end of the probes). (DOCX)

S6 Table Primers used for RNA:DNA hybrid detection. (DOCX)

S7 Table Primers used for detection of the T7 endonuclease I digested fragments. (DOCX)

S8 Table Primers used for 3C-PCR. (DOCX)

S9 Table Primers used for 3C-qPCR. (DOCX)

S10 Table Primers used for RNAi plasmid constructing. (DOCX)

S11 Table Primers used for cloning 1,671-bp CRM1 into pET-30a vector. (DOCX)

## Abbreviations

3C: chromatin conformation capture
AFM: atomic force microscopy
Alu: *Arthrobacter luteus*
BAC: bacterial artificial chromosome
CENH3: centromeric H3 variant
CENP-A: centromeric protein A
ChIP-seq: chromatin immunoprecipitation following high-throughput sequencing
CpG: cytosine-phosphate-guanine
CRM: centromeric retrotransposon
Cse4: Chromosome segregation protein 4
CTCF: CCCTC-binding factor
DRIP-seq: DNA-RNA immunoprecipitation sequencing
FACT: Facilitates Chromatin Transcription
FISH: fluorescence in situ hybridization
G1 phase: Gap 1 phase
GADD45A: growth arrest and DNA damage protein 45A
GEO: Gene Expression Omnibus
H3-Ser10: serine 10 in histone H3
IgG: Immunoglobulin G
IGV: Integrative Genome Viewer
input-seq: input sequencing
IP: immunoprecipitation
L1: LINE-1
lncRNA: long noncoding RNA
lncRNA-seq: lncRNA sequencing
LTR: long terminal repeat
nt: nucleotide
qPCR: quantitative PCR
rDNA: ribosomal DNA
RIP: RNA immunoprecipitation
RIP-seq: RIP sequencing
RNA-seq: RNA sequencing
RNAi: RNA interference
RNAPII: RNA Polymerase II
RPM: reads per million
RT-PCR: reverse transcription PCR
S phase: Synthesis phase
ssDNA: single-strand DNA
TCP-1/cpn60: chaperonin-60/T-complex protein
