# Supplementary figures and images for: Back-spliced RNA from retrotransposon binds to centromere and regulates centromeric chromatin loops in maize
Source: PLoS Biol. 2020 Jan 29;18(1):e3000582. doi: 10.1371/journal.pbio.3000582 (PMC7010299; doi:10.1371/journal.pbio.3000582)

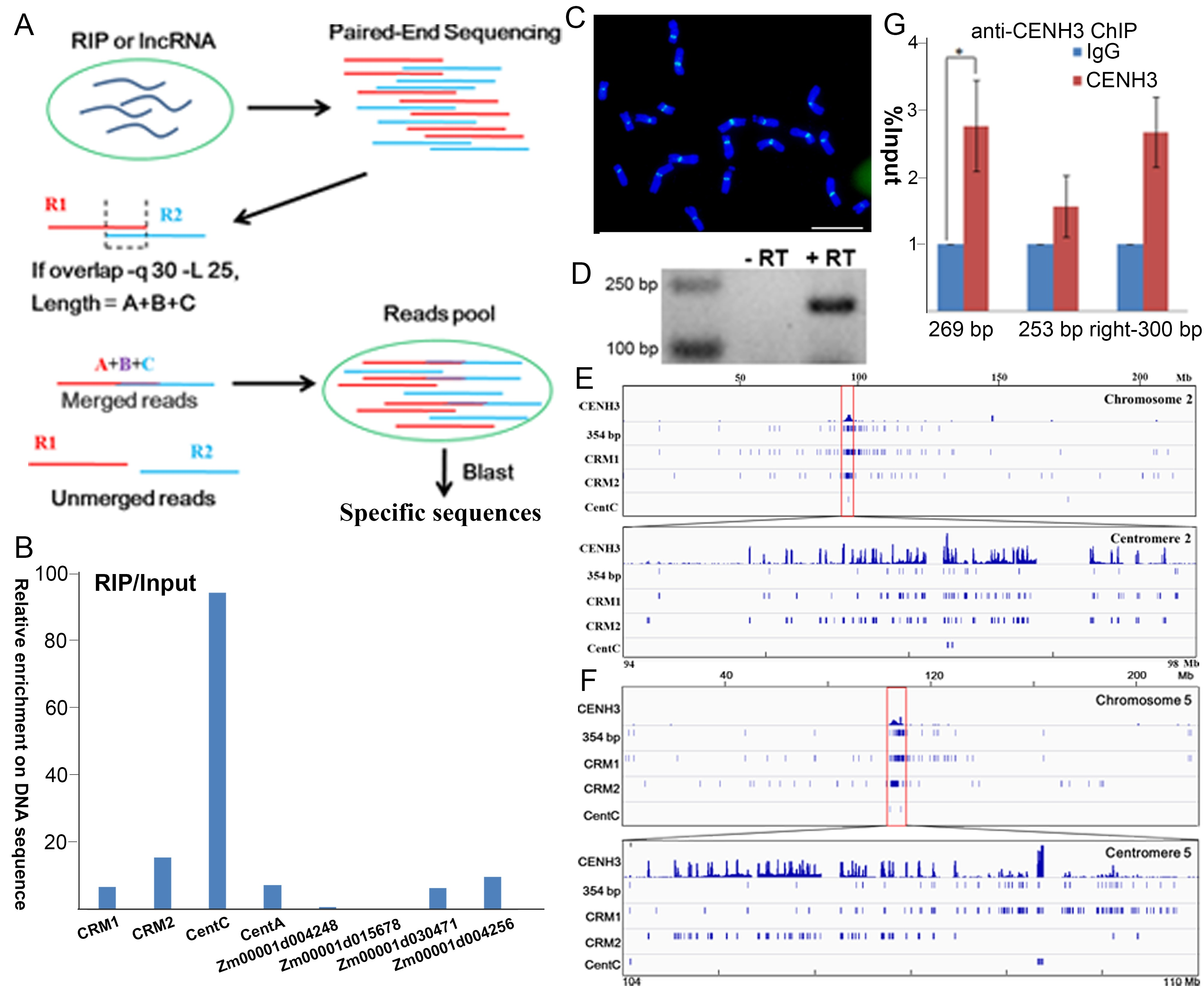

Supplement: S1 Fig — (A) The strategy of joining the paired-end reads for BLAST searching the centromere retrotransposons. (B) Relative enrichment of reads from the anti-CENH3 RIP-seq data associated with CRM elements, CentC repeats, 2 expressed centromeric genes (Zm00001d030471 and Zm00001d004256), and the 2 unexpressed genes (Zm00001d004248 and Zm00001d030471) located in centromere regions near CRM1 elements. The dotted line represents the value of no enrichment. (C) DNA-FISH of the 354-bp clone sequence. Blue indicates DAPI. Green indicates the sequence. Bar = 10 μm. (D) RT-PCR analysis of the 354-nt RNA without and with reverse transcription. (E and F) The distribution of the 354-bp sequence on Chromosome 2 (panel E) and Chromosome 5 (panel F). The x axis in the first panel represents the positions along Chromosome 2 (panel E) and Chromosome 5 (panel F). The x axis in the second panel represents the enlarged view of cen2 (panel E) and cen5 (panel F) as illustrated by CENH3 enrichment. The first track of each panel represents the centromeric region. The other 4 tracks represent the distributions of the 354-bp, CRM1, CRM2, and CentC, respectively. The red box indicates the centromeric region. The peak heights in each track represent the RPM value (0–1). (G) Anti-CENH3 ChIP-qPCR shows the enrichment of the 269-bp and 253-bp DNA in CENH3 binding regions. “right-300 bp” represents the 300-bp DNA on the right of the 607-bp sequence. Actin was used as an internal reference gene. The columns and error bars represent the relative value and standard error of the means (n = 3), respectively. P values were determined by Student t test: *P < 0.05, **P < 0.01. The data underlying this figure can be found in S1 Data, S1 Raw Images, and on Github (https://github.com/sxx-ying/maize-centromere-circRNA). (TIF) [file pbio.3000582.s001.tif]

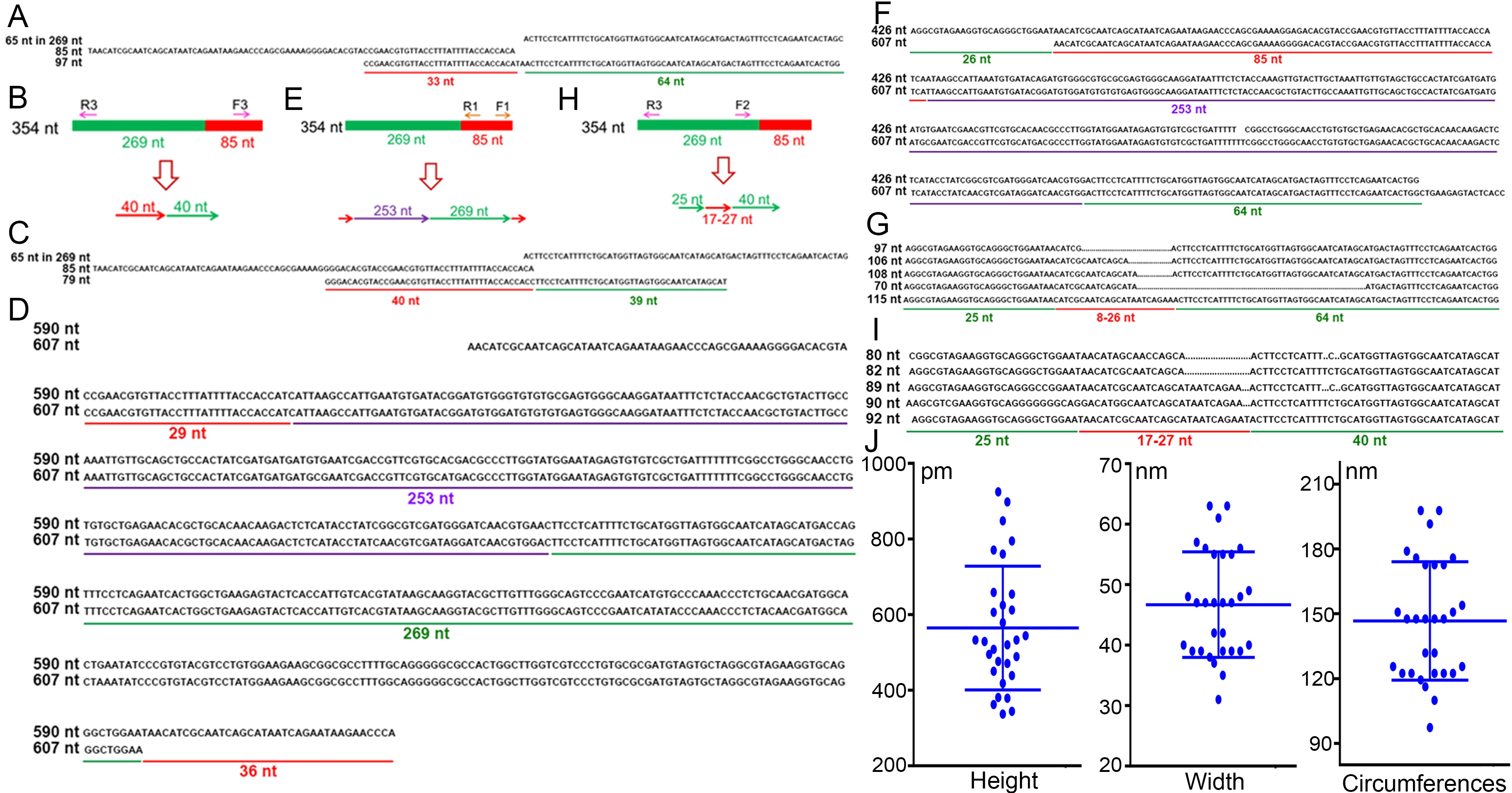

Supplement: S2 Fig — (A) The sequences amplified using primers F4+R2. The first 2 lines represent 65-nt sequence from the 269-nt region and the 85-nt sequence, respectively. The third line represents the amplified 97-nt sequence. (B) Divergent primers F3+R3 were used to detect the existence of the 354-nt circular RNA. (C) The sequences amplified using primers F3+R3. In (A) and (C), the upper 2 lines are 65-nt sequence in the 269-nt and 85-nt sequence, respectively. The third line is the amplified sequence. (D) The sequences amplified using primers F2+R2. The first line represents the amplified 590-nt sequence, and the second line represents the 607-nt sequence. (E) Divergent primers F1+R1 were used to detect the existence of the 607-nt circular RNA. (F) The sequences amplified using primers F1+R1. The first line is the amplified sequence and the second line is the 607-nt sequence. (G) The shorter sequences amplified with primers F2+R2. Five sequences are shown. (H) Divergent primers F2+R3 confirmed the existence of the 277- to 296-nt circular RNA. The 17- to 27-nt sequence from the 85 nt was connected to the 5′ end of the 269-nt sequence in the amplified sequences. In (B), (E), and (H), the upper panel represents the positions of primers on the 354-nt sequence, and the lower panel represents the amplified sequences. (I) The sequences amplified with primers F2+R3. Five sequences are shown. The green bars and green lines represent the 269-nt sequence in the 354-nt and the amplified sequence, respectively. The red bars and red lines represent the 85-nt sequence in the 354-nt and the amplified sequence, respectively. The purple lines represent the intermediate 253-nt sequence. (J) Distribution of the heights, widths, and circumferences of the circular RNAs. Each point indicates a circular RNA. About 30 molecules are calculated. Mean and standard error of mean indicated. The data underlying this figure can be found in S1 Data. (TIF) [file pbio.3000582.s002.tif]

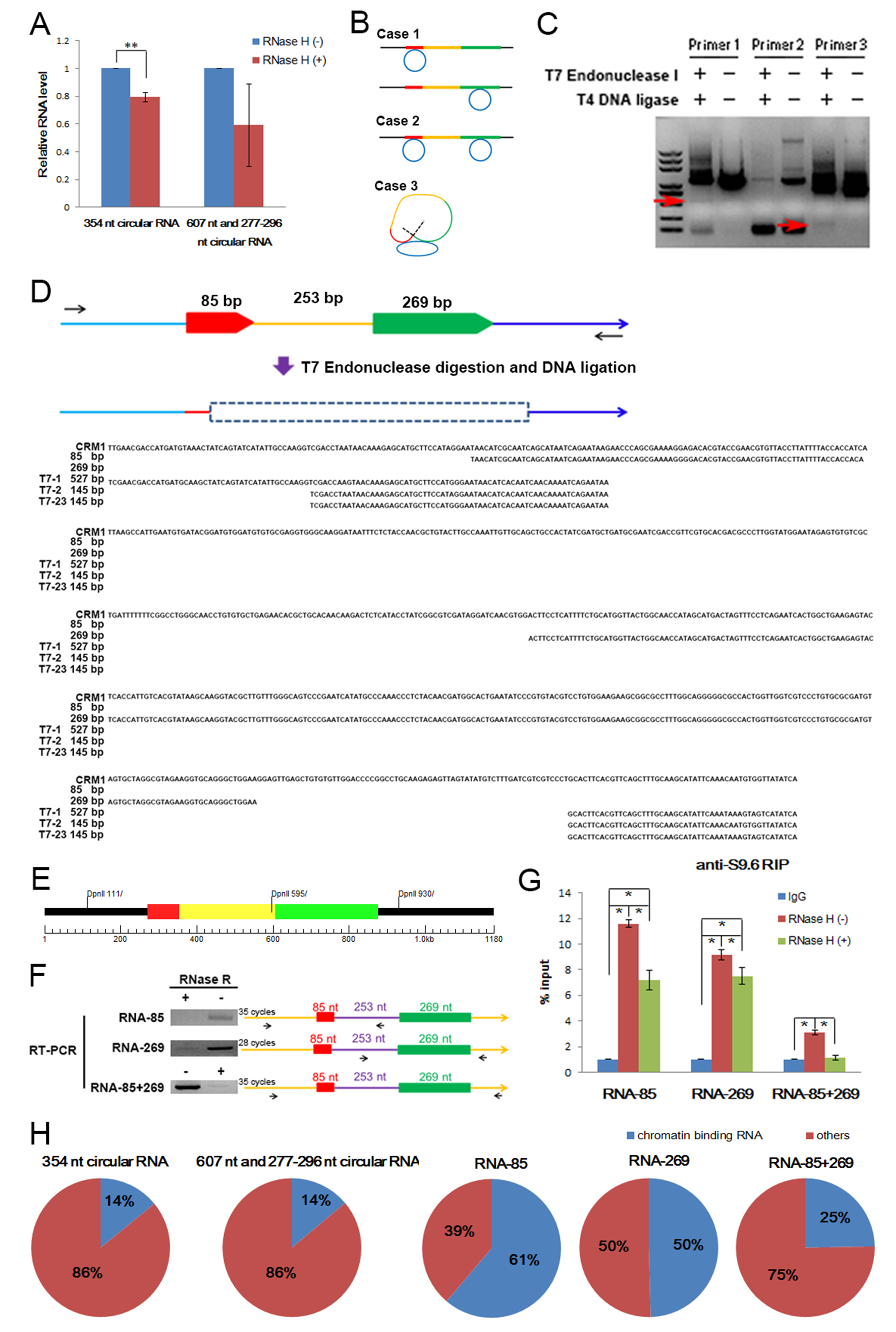

Supplement: S3 Fig — (A) RNA:DNA hybrids formed by circular CRM1 RNAs were checked by RNase H treatment. Chromatin-binding RNA was used for confirmation. (B) The potential cases for R-loop formation by circular CRM1 RNAs. The blue circles represent circular CRM1 RNAs. (C) Detection of the R-loop structure by T7 endonuclease I digestion and subsequent ligation. The red arrows show the shortened sequences. (D) Shortened sequence (showed by the rectangle with dotted line) obtained after T7 endonuclease I treatment and DNA ligation. The arrows on 2 sides show the primer positions. The detailed sequences of the shorter PCR bands were shown in the lower panel. The upper 3 tracks show the sequences of CRM1, the 85 bp, and the 269 bp, respectively. The fourth track shows the shorter sequence amplified by Primer 1. The last 2 tracks show the shorter sequences amplified by Primer 3. (E) The DpnII digestion sites on the 607-bp region and the surrounding regions. (F) RNA-85, RNA-269, and RNA-85+269 were sensitive to RNase R treatment. The right panel shows the positions of the primers. (G) Anti-S9.6 RIP-qPCR was used to confirm the R-loop formation by linear CRM1 RNAs. Chromatin-binding RNA was used for RIP. (H) The percentages of chromatin-binding RNA in the total RNA for the CRM1 RNAs. In (A) and (G), Actin was used as an internal reference gene, the columns and the error bars represent the relative value and standard error of the means (n = 3). P values were determined by Student t test: *P < 0.05, **P < 0.01. In (B), (D), (E), and (F), the red, yellow, and green bars represent the 85-bp, 253-bp, and 269-bp region, respectively. The black lines represent the left and right sides of the 607-bp region. The data underlying this figure can be found in S1 Data and S1 Raw Images. (TIF) [file pbio.3000582.s003.tif]

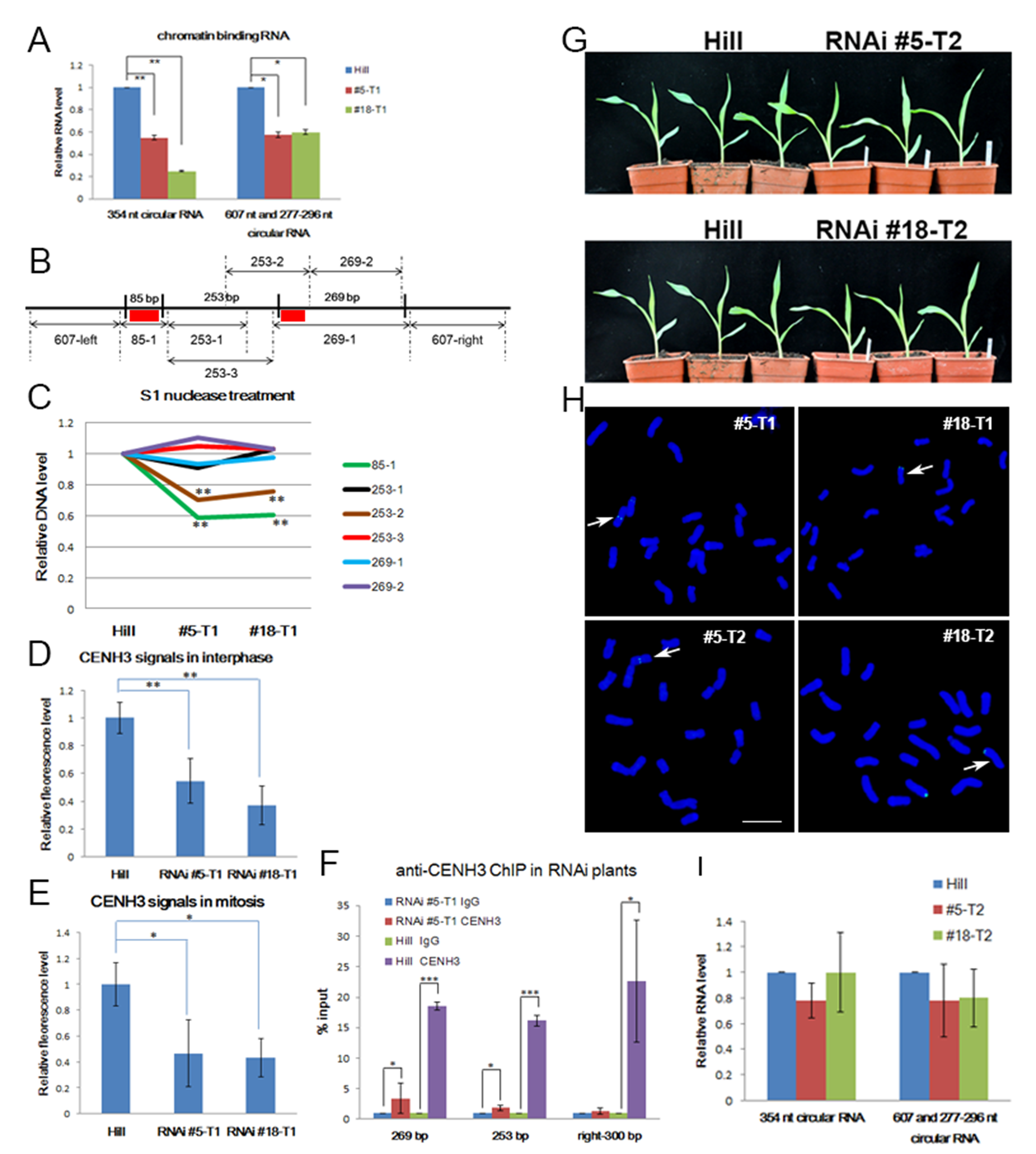

Supplement: S4 Fig — (A) RT-qPCR shows that chromatin-binding levels of the 354-nt, 607-nt, and 277- to 296-nt circular RNAs are reduced in the RNAi plants. (B) The regions chosen for detecting the ssDNA sites are marked as 85–1, 253–1, 253–2, 253–3, 269–1, and 269–2. The red bars show the ssDNA regions confirmed in (C). (C) The ssDNA sites confirmed by 1,000 U S1 nuclease treatment. The DNA with no S1 nuclease treatment was used as the control template; 607-left was used as an internal reference gene. (D and E) Quantification of the CENH3 fluorescence intensity in the T1 generation of RNAi plants and HiII. Thirty cells from interphase (D) and 15 cells in mitosis (E) were measured to quantify the CENH3 signals. (F) Anti-CENH3 ChIP-qPCR analysis showed reduced CENH3 localization in CRM1 regions in the T1 generation of RNAi plants 5. (G) Seedling phenotypes of the T2 generation of the RNAi plants. The left 3 seedlings are from HiII and the right 3 seedlings are from the T2 generation plants in each panel. (H) FISH detection of the RNAi vector in the T1 and T2 generation of the RNAi plants. Blue indicates DAPI. Green indicates the vector signals. Bar = 10 μm. (I) The RNA levels of CRM1 circular RNAs in the T2 generation of RNAi plants. In (A), (C), (D), (E) and (I), HiII is the control. In (A), (F), and (I), Actin was used as an internal reference gene. In (A), (C), (D), (E), (F), and (I), the columns and error bars represent the relative values and standard error of the means, respectively. P values were determined by Student t test: *P < 0.05, **P < 0.01. The data underlying this figure can be found in S1 Data. (TIF) [file pbio.3000582.s004.tif]

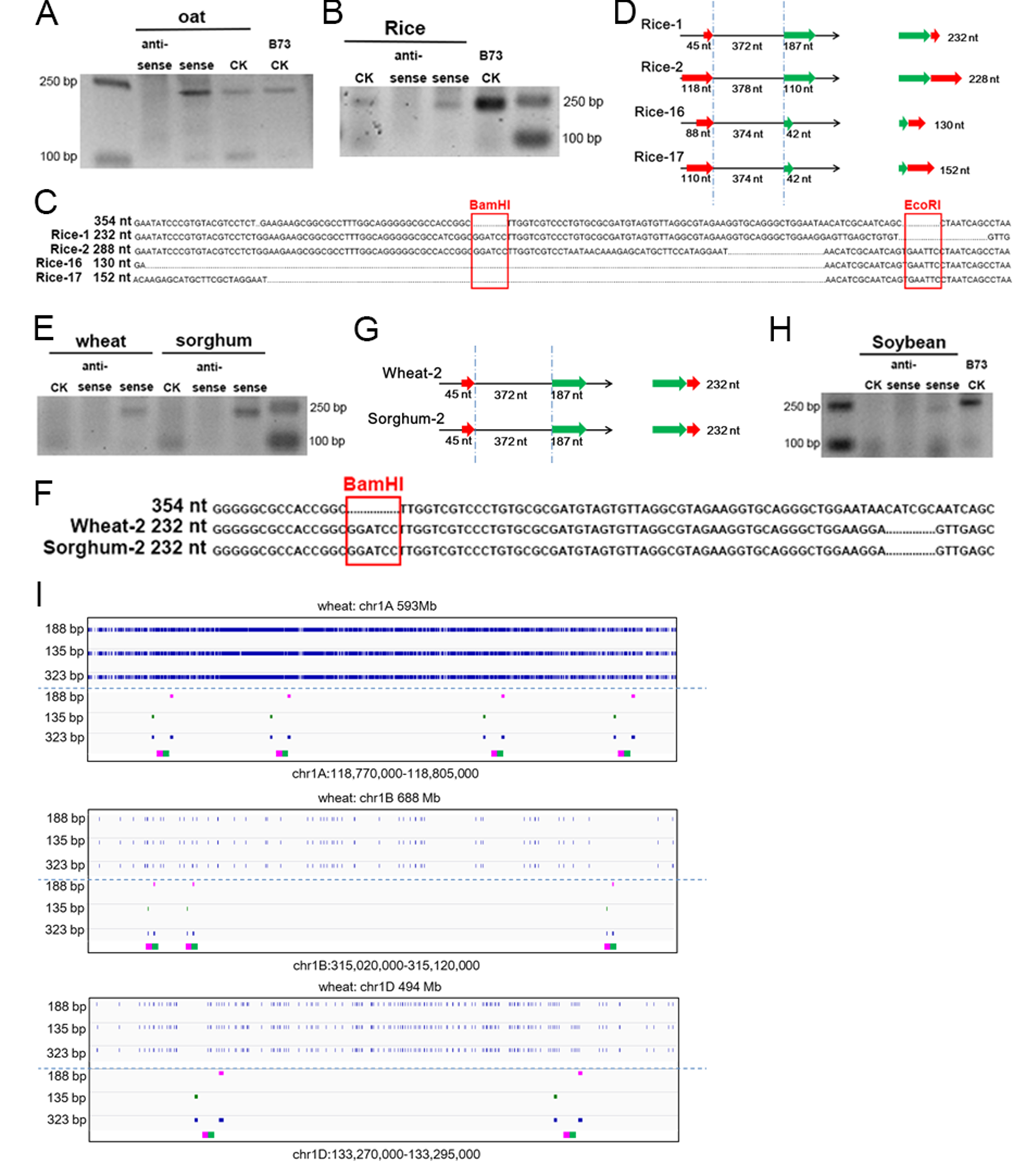

Supplement: S5 Fig — (A, B, E, and H) The sense-strand–transcribed CRM1 RNA can be spliced into the 354-nt–like back-spliced RNA after being transformed into oat (A), rice (B), wheat and sorghum (E), and soybean (H) protoplasts. (C and F) The 354-nt–like back-spliced RNAs from rice (C) and wheat and sorghum (F) protoplast transformation. The red frames mark the labeled digestion sites. The first track shows the 354-nt RNA and the other 3 tracks show the back-spliced sequences in protoplasts. (D and G) Detailed process of the back splicing in rice (D) as well as wheat and sorghum (G) protoplast. The red arrows show the upstream sequence, and the green arrows show the downstream sequence. The left panel shows the sequence positions on the 1,671-nt sequence, and the right panel shows the final spliced sequences. (I) The distributions of the 323-bp sequence (consisted of 188-bp and 135-bp sequence) in the AA, BB, and DD subgenome of common wheat. The x axes in each panel represent positions along Chromosome 1A, 1B, and 1D. The first 3 tracks of each panel represent distributions of the 323-bp, 188-bp, and 135-bp sequence along the whole chromosome. The other 3 tracks represent several detailed positions. The data underlying this figure can be found in S1 Data, GSE137701, and on Github (https://github.com/sxx-ying/maize-centromere-circRNA). (TIF) [file pbio.3000582.s005.tif]
