# Supplementary material for: Back-spliced RNA from retrotransposon binds to centromere and regulates centromeric chromatin loops in maize
Source: PLoS Biol. 2020 Jan 29;18(1):e3000582. doi: 10.1371/journal.pbio.3000582 (PMC7010299; doi:10.1371/journal.pbio.3000582)
Supplement: S1 Raw Images — (PDF) [file pbio.3000582.s006.pdf]

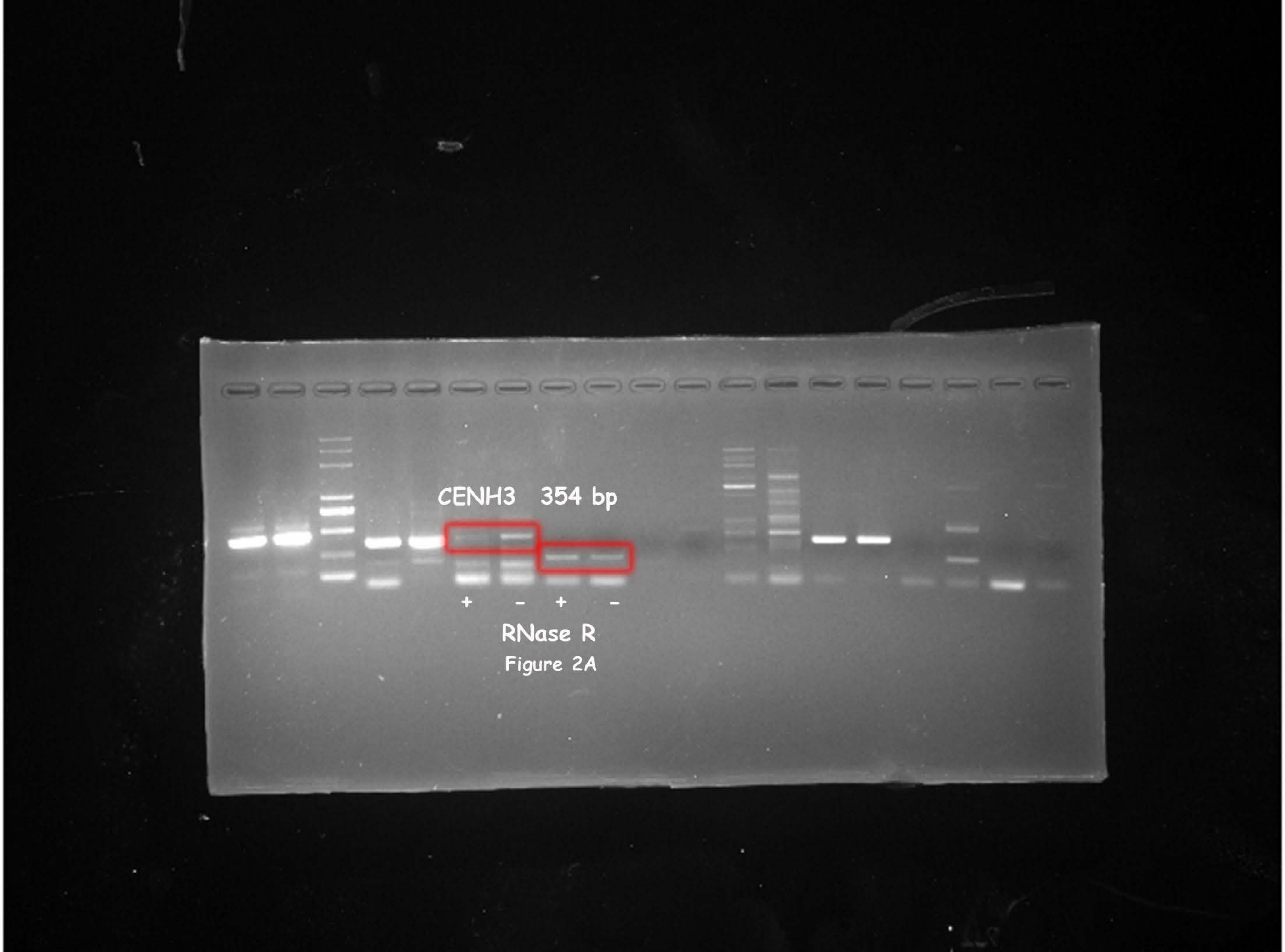

Figure 2A

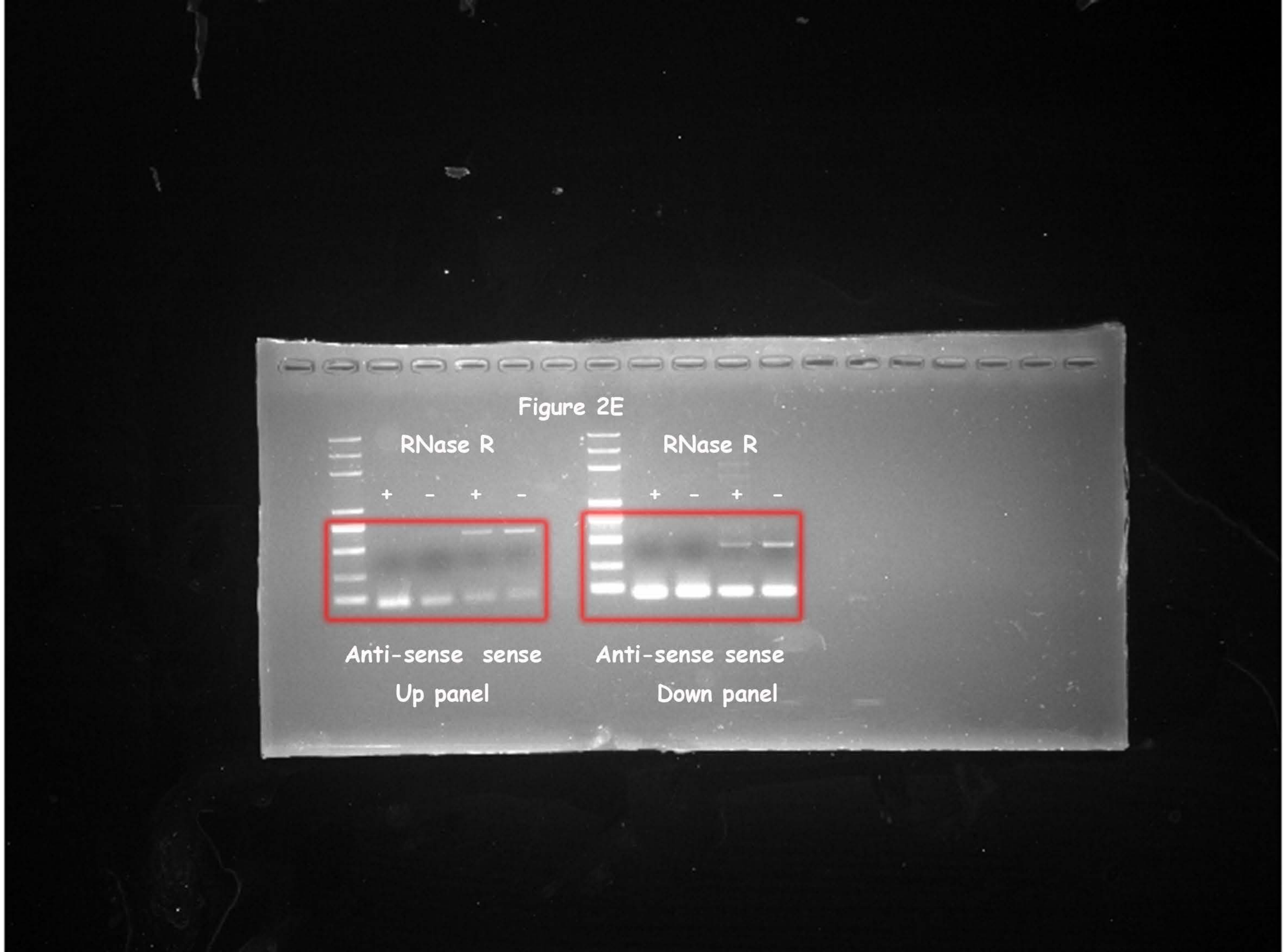
Figure 2E
RNase R
RNase R
+ - + -
+ - + -
Anti-sense sense
Anti-sense sense
Up panel
Down panel

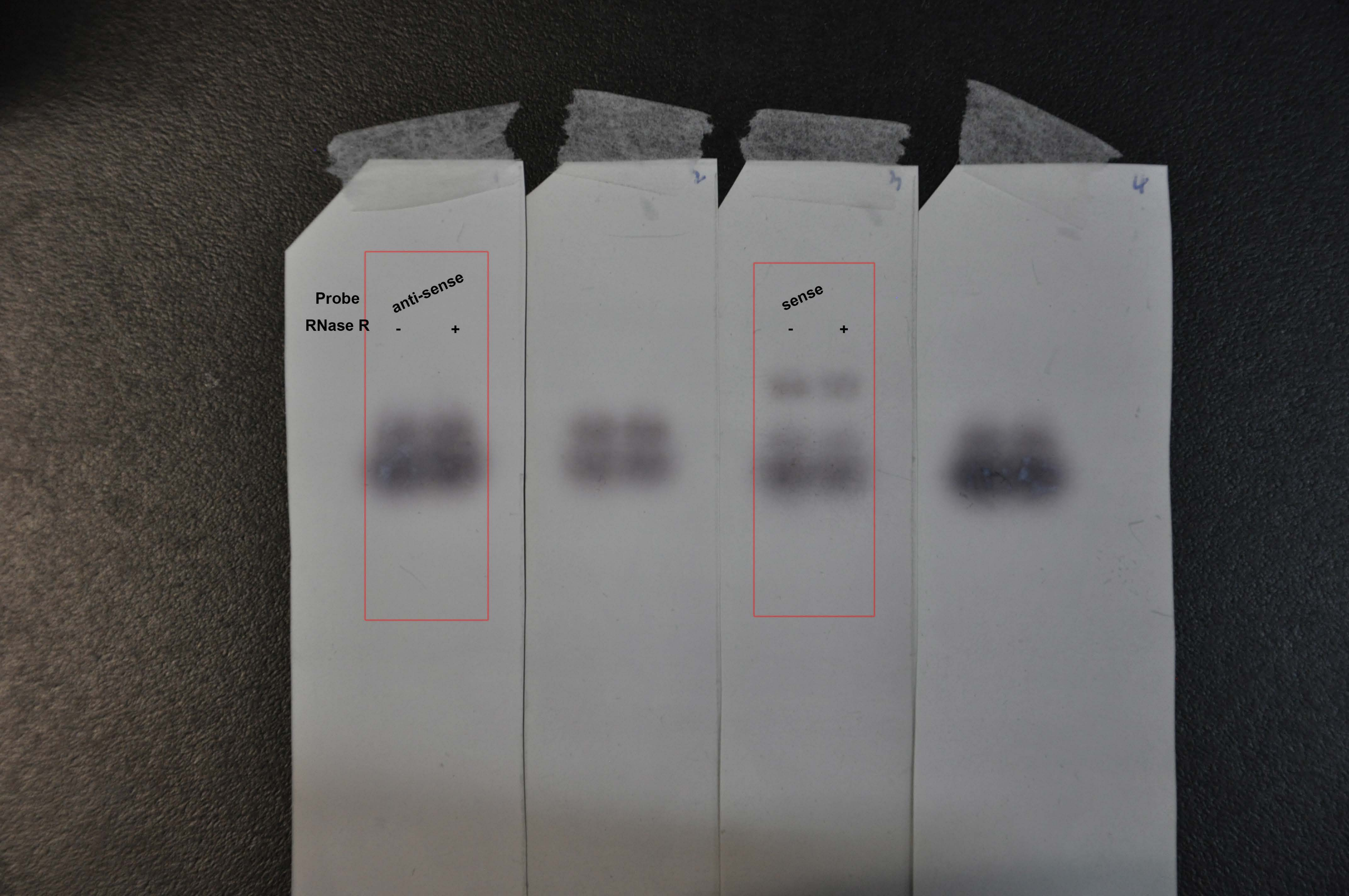

Probe
anti-sense
RNase R
-
+
sense
-
+

Fig 2. Detection of the full-length circular CRM1 RNAs.

(A) RNase R treatment of *Cenh3* mRNA and 354-nt RNA. The first two wells marked by the red box show the changes of *Cenh3* mRNA with and without RNase R treatment. The third and forth wells show the changes of 354-nt mRNA with and without RNase R treatment. (E) Divergent PCR showed that the 607-nt and 277- to 296- nt RNAs were stable after RNase R treatment. The first and third wells in each panel show the RNA samples with RNase R treatment, and the second and forth wells show the samples without RNase R treatment. The first two samples in each panel were from the RNA purified by biotinylated anti-sense probes, and the other two were from RNA purified by biotinylated sense probes. The larger bands in each panel show the 607-nt RNA. The smaller bands in the right panel show the 277- to 296- nt RNAs. (F) A northern blot was performed using digoxin-labeled 25-bp antisense or sense probes. The probe was located in the 269-nt region. The RNA was run in 3% denaturing formaldehyde agarose gel. The first two columns show the RNA samples purified by biotinylated anti-sense oligos. The fifth and sixth columns show the RNA samples purified by biotinylated sense oligos. The first and fifth samples were without RNase R treatment and the second and sixth were with RNase R treatment.

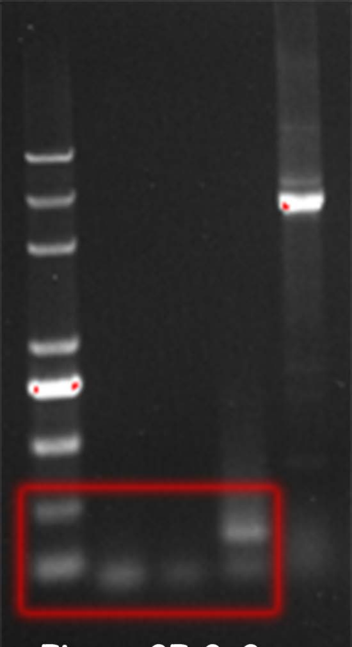

Figure 3F 2+3

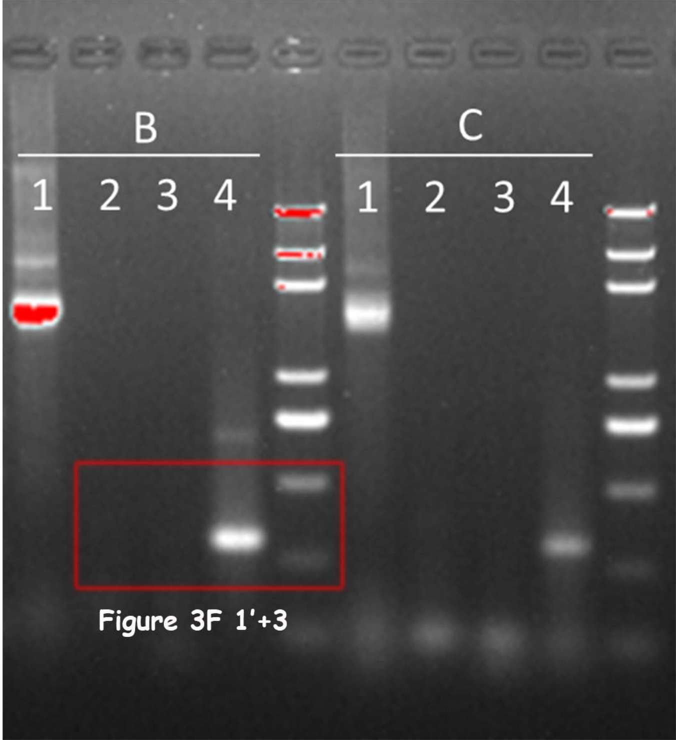
B
1 2 3 4
C
1 2 3 4
Figure 3F 1'+3

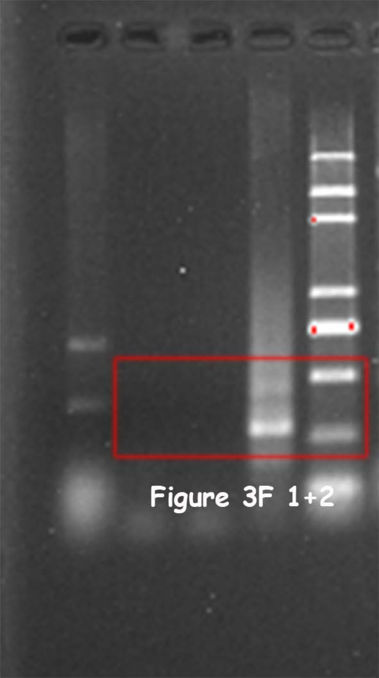
Figure 3F 1+2

Fig 3. Circular CMR1 RNAs induce chromatin loops in the centromere.

(F) 3C-PCR confirms the potential ligations of chromatin loops after *DpnII* digestion. The first image shows the PCR results in the undigested, unligated samples and 3C samples under potential ligation form of ② and ③ (marked in Fig 3D and 3E). The left panel (marked by the red box) in the second image shows the PCR results in the undigested, unligated samples and 3C samples under potential ligation form of '①' and ③ (marked in Fig 3D and 3E). The third image shows the PCR results in the undigested, unligated samples and 3C samples under potential ligation form of ① and ② (marked in Fig 3D and 3E).

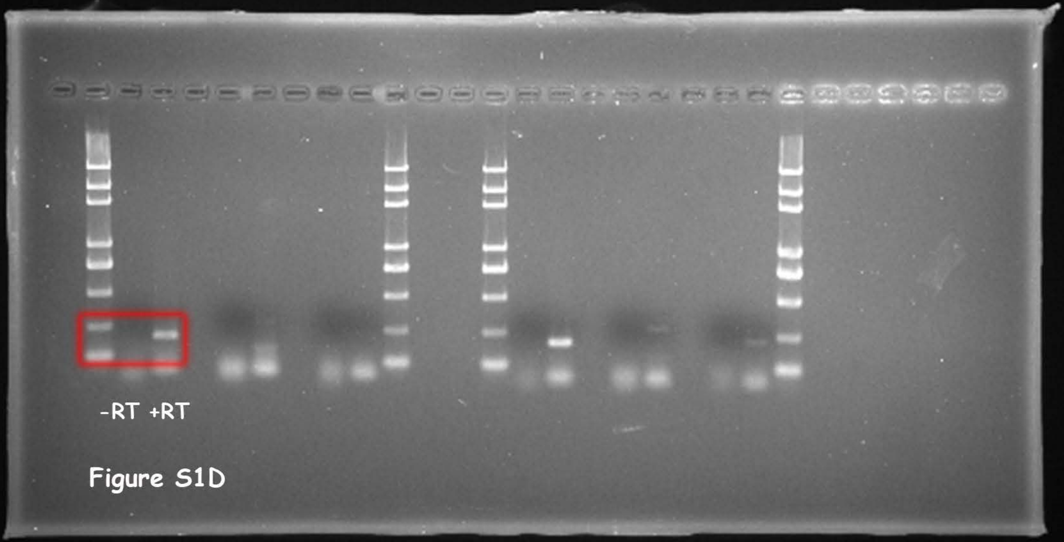

Figure S1D

S1 Fig. Back-spliced RNA from CRM1 in the centromere.

(D) RT-PCR analysis of the 354-nt RNA. The first and second samples in the red box were without and with reverse transcription, respectively.

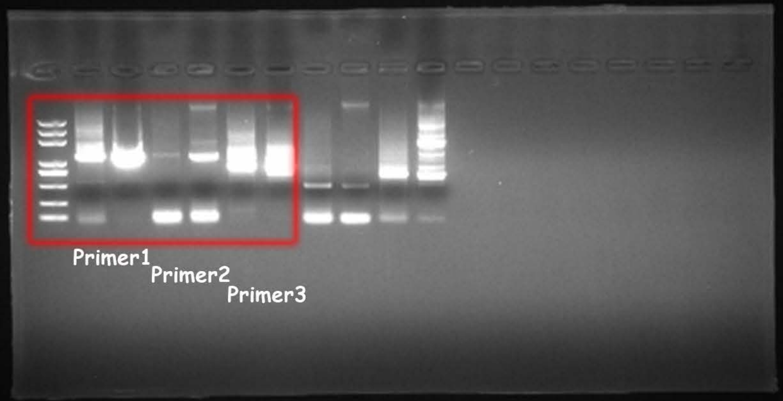

Figure S3C

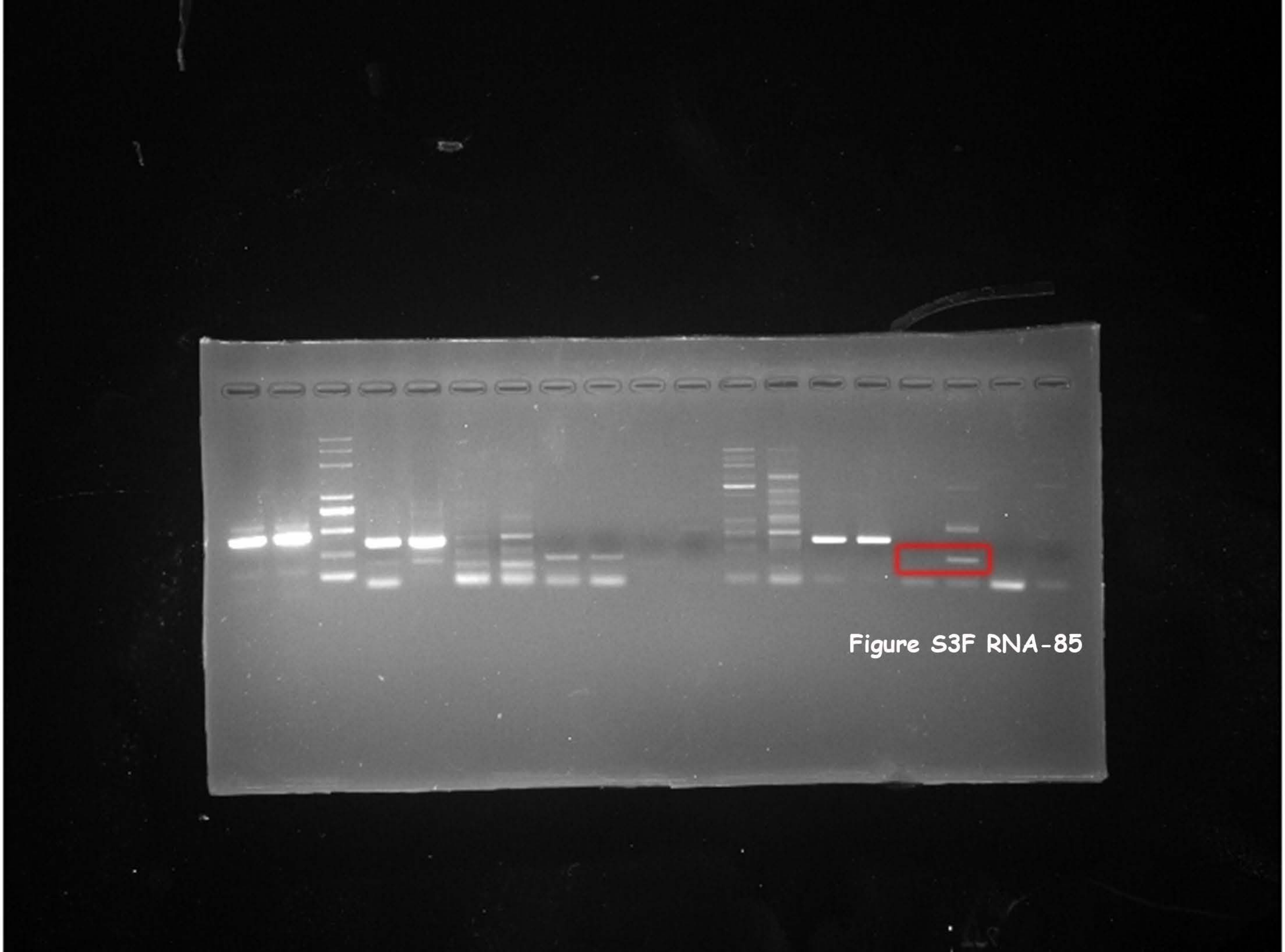

Figure S3F RNA-85

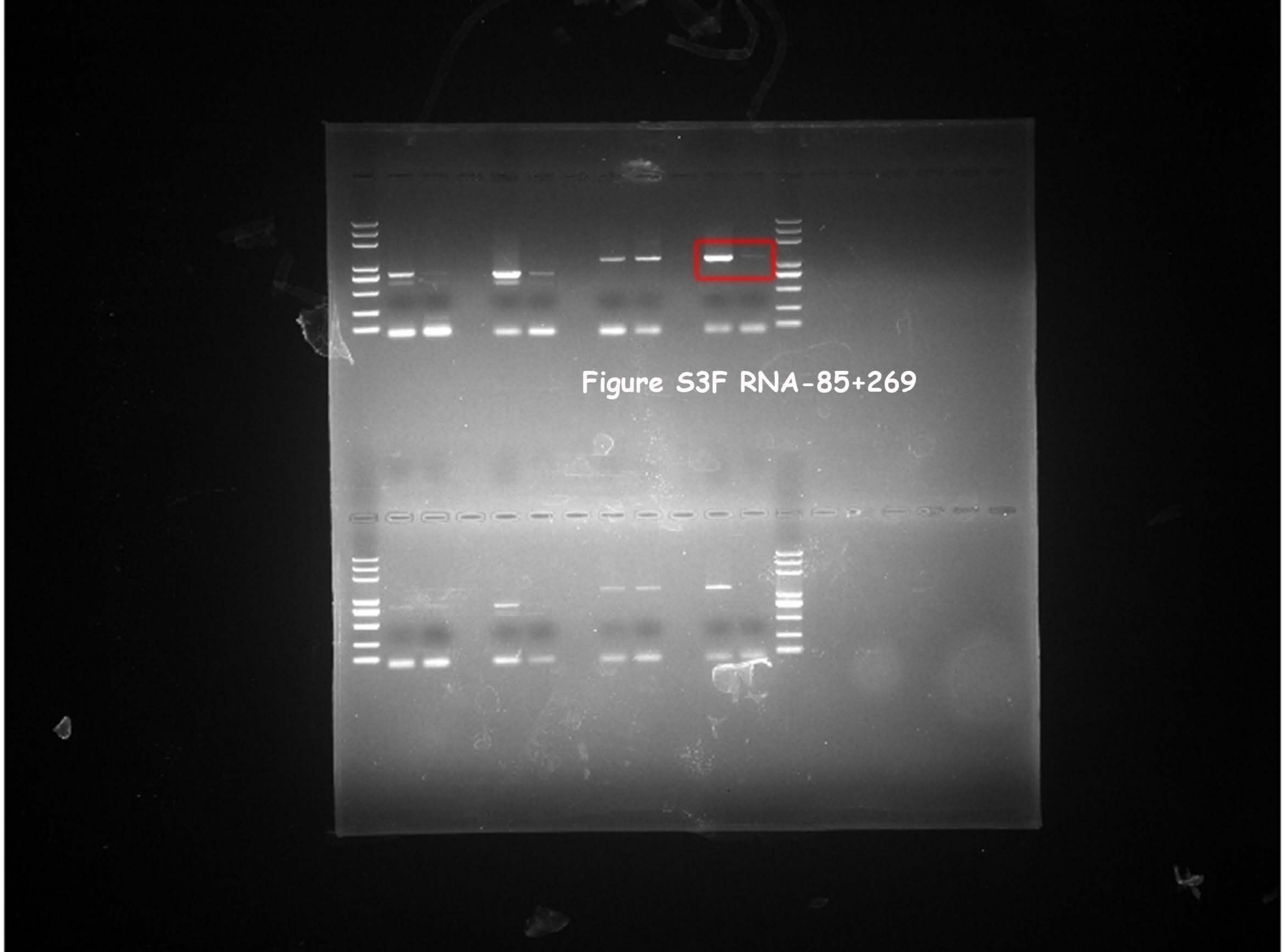
Figure S3F RNA-85+269

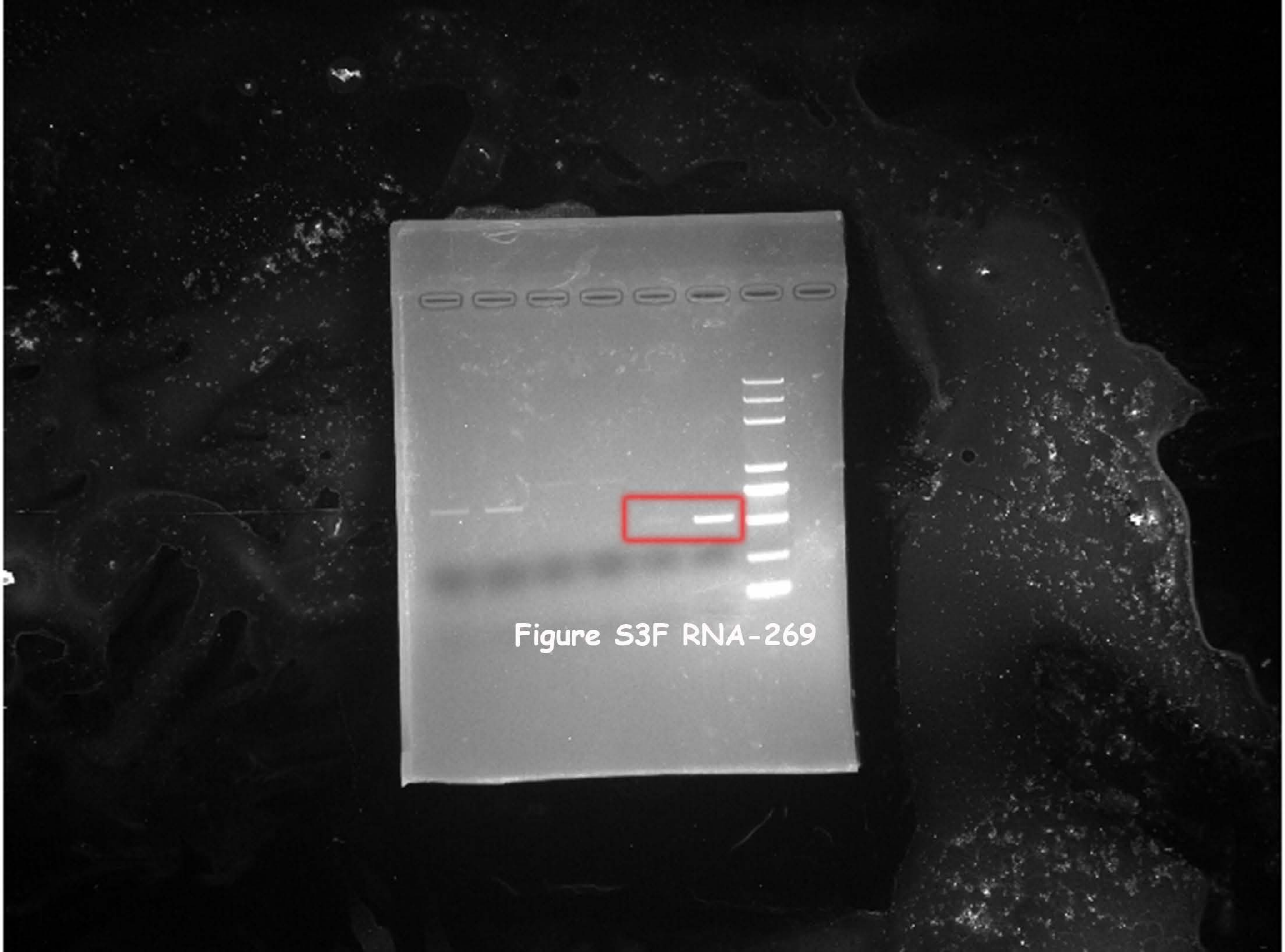
Figure S3F RNA-269

S3 Fig. Circular CRM1 RNAs induce chromatin loops in the centromere.

(C) Detection of the R-loop structure by T7 endonuclease I digestion and subsequent ligation. Three pairs of primers were used for PCR. For each pair of primer, the T7 endonuclease I digested and T4 DNA ligase ligated sample was shown in the first well, and the sample with no treatment was in the well afterwards. Additional bands can be found in the T7 endonuclease I digested samples from the PCR reactions using Primer 1 and 3. (F) RNA-85, RNA-269 and RNA-85+269 were sensitive to RNase R treatment. The first image shows the changes of RNA-85 after RNase R treatment. The first well marked in the red box shows the sample with RNase R treatment and the second well shows the sample without RNase R treatment. The smaller band from the second well represents RNA-85. The second image shows the changes of RNA-269 after RNase R treatment. The first well marked in the red box shows the sample with RNase R treatment and the second well shows the sample without RNase R treatment. The third image shows the changes of RNA-85+269 after RNase R treatment. The first well marked in the red box shows the sample without RNase R treatment and the second well shows the sample with RNase R treatment.

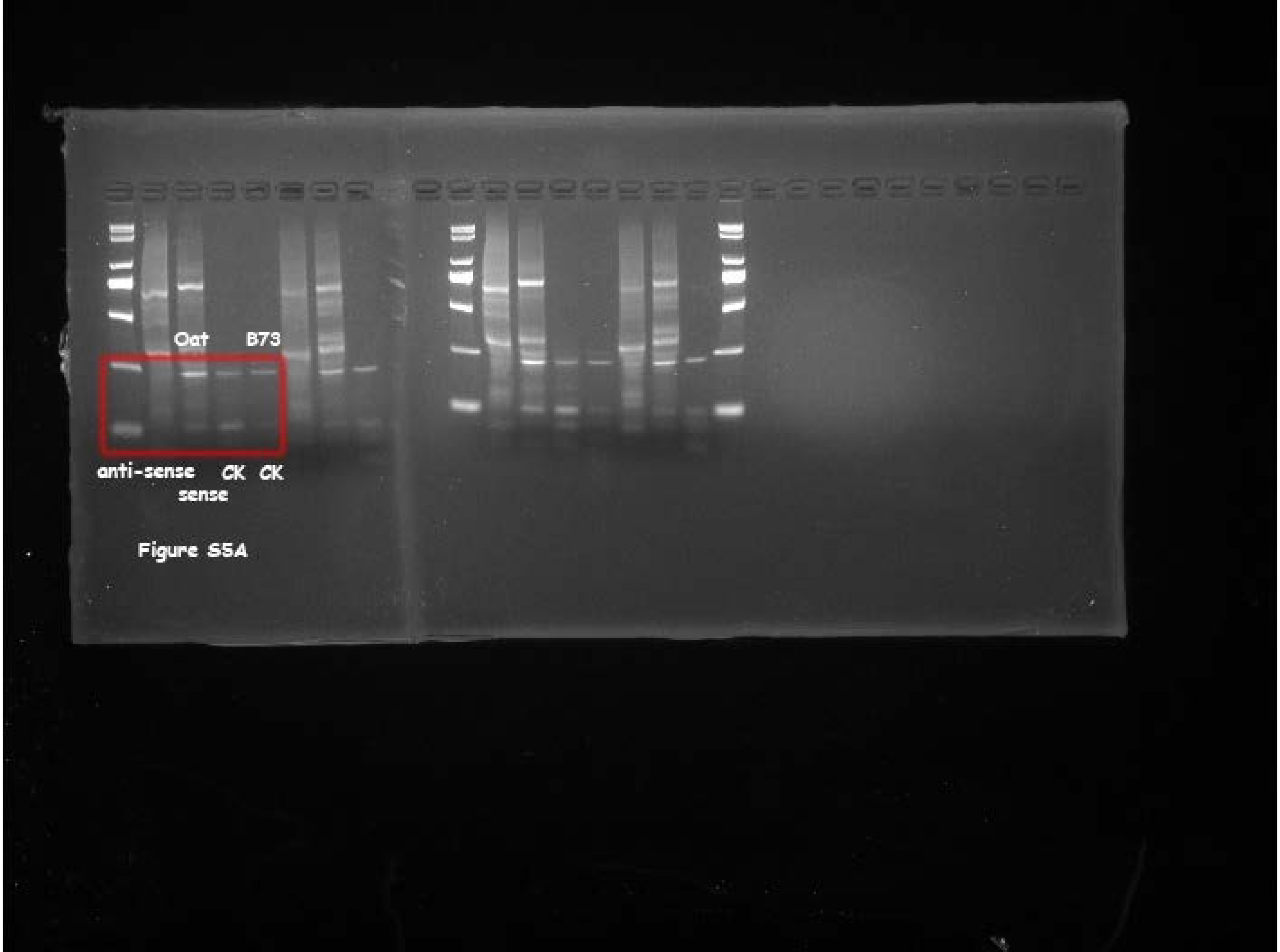

Figure S5A

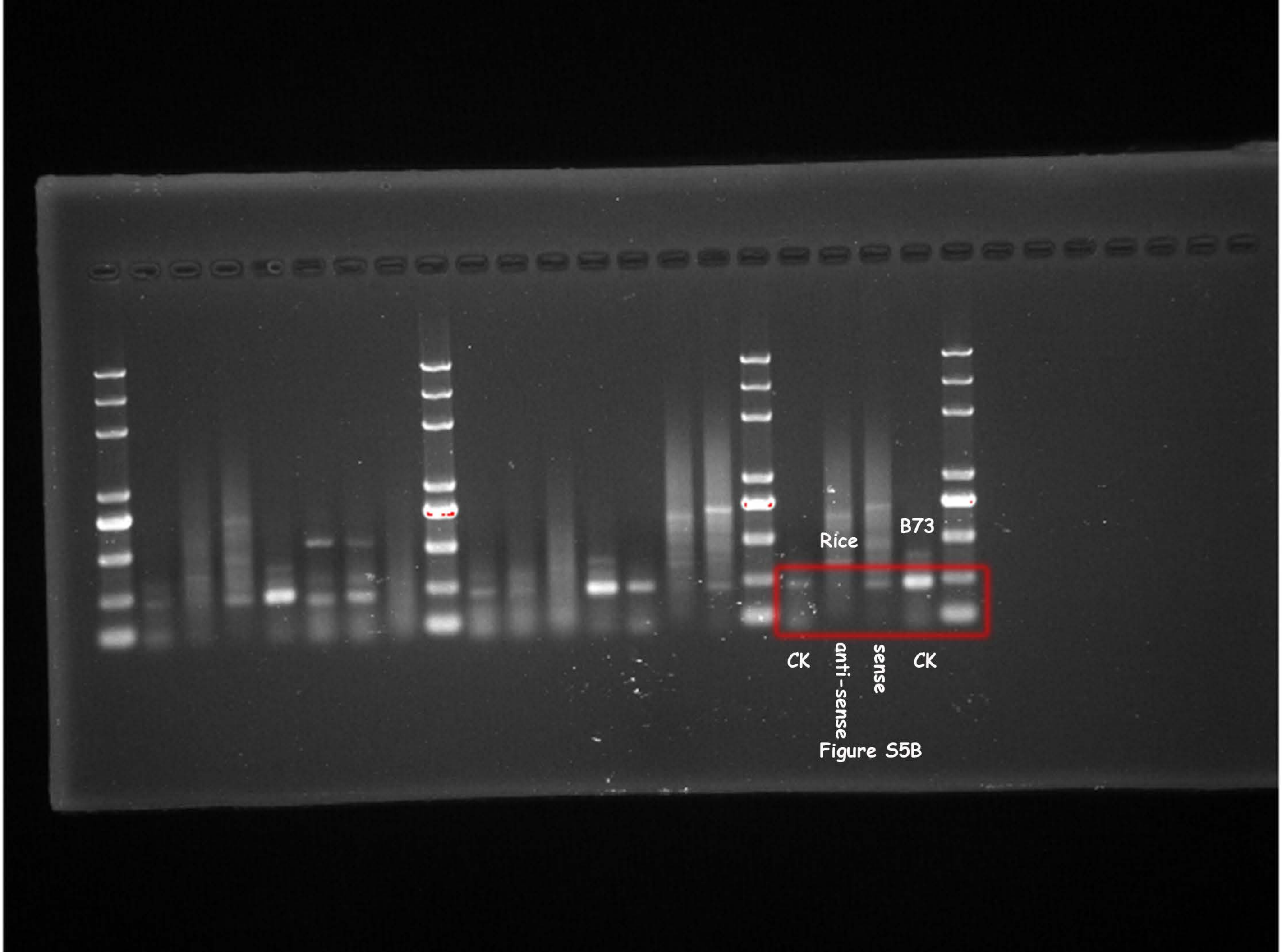
Rice
B73
CK
anti-sense
sense
CK
Figure S5B

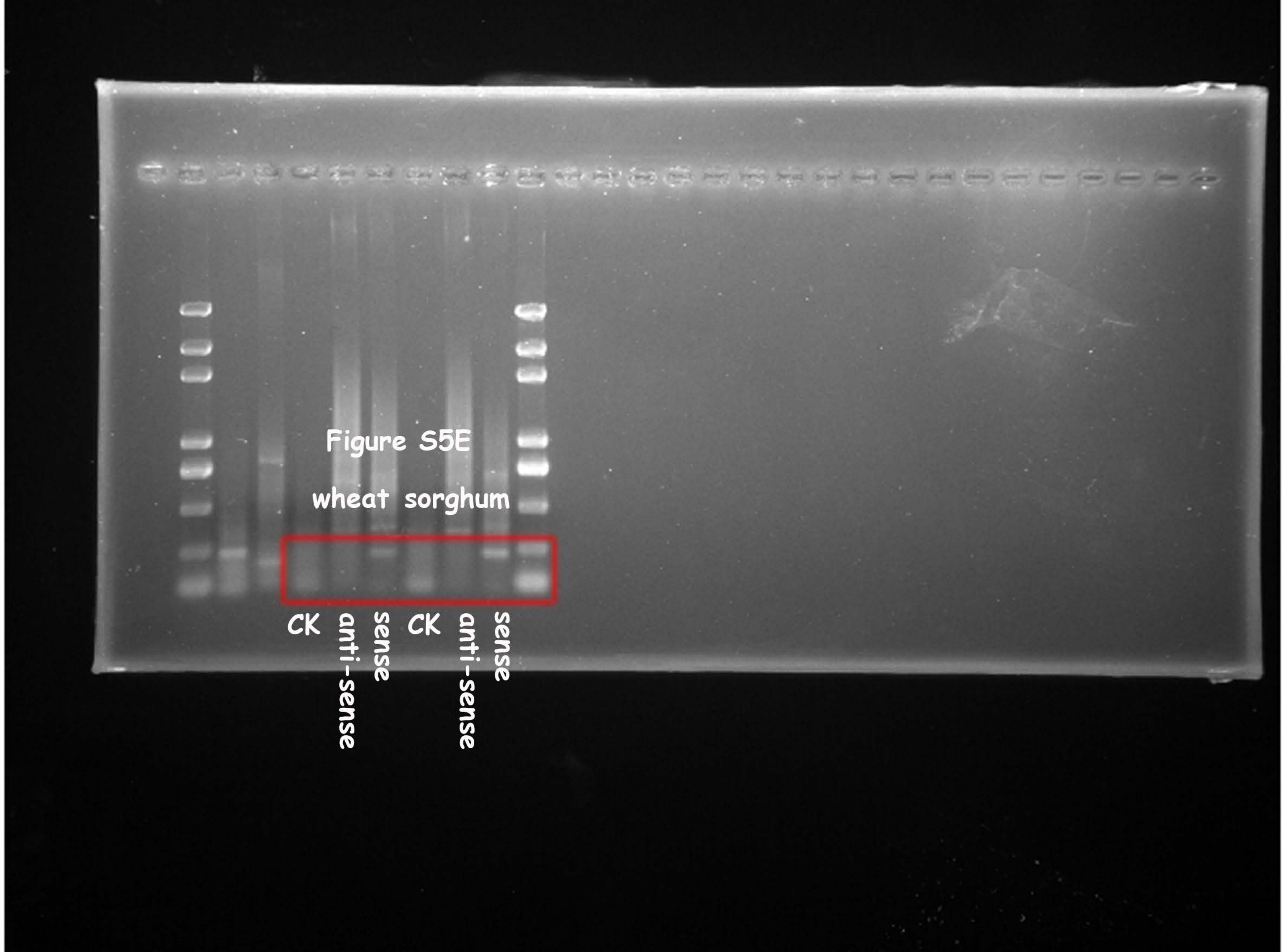

Figure S5E
wheat sorghum
CK
anti-sense
sense
CK
anti-sense
sense

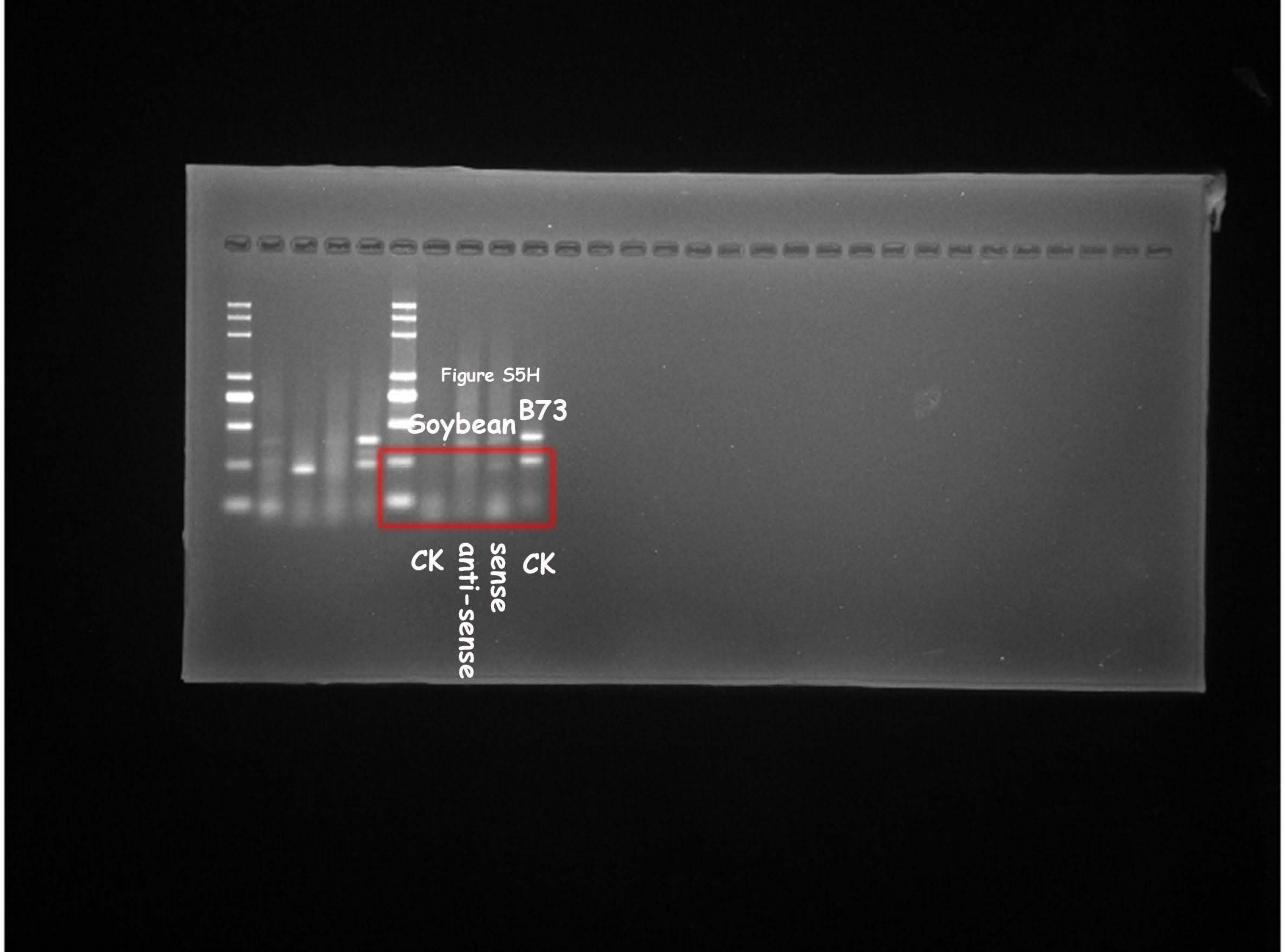
Figure S5H
Soybean
B73
CK
anti-sense
sense
CK

S5 Fig. The back-splicing process in retrotransposon is conserved in crops.

(A) The sense strand transcribed CRM1 RNA can be spliced into the 354-nt-like back-spliced RNA after being transformed into oat protoplasts. The four samples marked in the red box were listed as oat protoplasts transformed with anti-sense CRM1 RNA (the first sample), oat protoplasts transformed with sense CRM1 RNA (the second sample), oat protoplasts transformed with water (the third sample), and B73 cDNA (the forth sample). (B) The sense strand transcribed CRM1 RNA can be spliced into the 354-nt-like back-spliced RNA after being transformed into rice protoplasts. The four samples marked in the red box were listed as rice protoplasts transformed with water (the first sample), rice protoplasts transformed with anti-sense CRM1 RNA (the second sample), rice protoplasts transformed with sense CRM1 RNA (the third sample), and B73 cDNA (the forth sample). (E) The sense strand transcribed CRM1 RNA can be spliced into the 354-nt-like back-spliced RNA after being transformed into wheat and sorghum protoplasts. The six samples marked in the red box were listed as wheat protoplasts transformed with water (the first sample), wheat protoplasts transformed with anti-sense CRM1 RNA (the second sample), wheat protoplasts transformed with sense CRM1 RNA (the third sample), sorghum protoplasts transformed with water (the forth sample), sorghum protoplasts transformed with anti-sense CRM1 RNA (the fifth sample), and sorghum protoplasts transformed with sense CRM1 RNA (the sixth sample). (H) The sense strand transcribed CRM1 RNA can be spliced into the 354-nt-like back-spliced RNA after being transformed into soybean protoplasts. The four samples marked in the red box were listed as soybean protoplasts transformed with water (the first sample), soybean protoplasts transformed with anti-sense CRM1 RNA (the second sample), soybean protoplasts transformed with sense CRM1 RNA (the third sample), and B73 cDNA (the forth sample).
