# Supplementary material for: Back-spliced RNA from retrotransposon binds to centromere and regulates centromeric chromatin loops in maize
Source: PLoS Biol. 2020 Jan 29;18(1):e3000582. doi: 10.1371/journal.pbio.3000582 (PMC7010299; doi:10.1371/journal.pbio.3000582)
Supplement: S1 Table — (DOCX) [file pbio.3000582.s008.docx]

**S1 Table. Primers used for PCR detection**

| Name | Sequence |
| --- | --- |
| 354 nt-p1-F | 5'TCCCGAATCATGTGCCCAAAC 3' |
| 354 nt-p1-R | 5'TGTGGTGGTAAAATAAAGGTAAC3' |
| M13-F | 5'AGCGGATAACAATTTCACACAGG 3' |
| M13-R | 5'GTCGTGACTGGGAAAACCCTGG 3' |
| *Cenh3*-F | 5'CCGGAAAGGGCTGCTGGGAC 3' |
| *Cenh3*-R | 5'CCAACGCCTTCCTCCGATAC 3' |
| RNA-85-p1-F | 5'CGACCTAATAACAAAGAGCATG 3' |
| RNA-85-p1-R | 5'CAGGTTGCCCAGGCCGAAAA 3' |
| RNA-269-p1-F | 5'TGGAATAGAGTGTGTCGCTGA 3' |
| RNA-269-p1-R | 5'GGGTATGCTTCGTTGTGGTTTAGTTGAG 3' |
| RNA-85+269-p1-F | 5'TCGCGAGCATACAAGTGCATAAC 3' |
| RNA-85+269-p1-R | 5'TGAGGATTATTACCAGGAGTTAC 3' |
| Wheat 323 nt-F  Wheat 323 nt-R | 5'AGCGGTAACATAGCCAAACAACGG 3'  5'AGTCACTTGCTGCAGCTCCACATCATAC 3' |
| DOP-PCR primer | 5'CCgACTCgAgNNNNNNATgTgg 3' |
