# Supplementary material for: Back-spliced RNA from retrotransposon binds to centromere and regulates centromeric chromatin loops in maize
Source: PLoS Biol. 2020 Jan 29;18(1):e3000582. doi: 10.1371/journal.pbio.3000582 (PMC7010299; doi:10.1371/journal.pbio.3000582)
Supplement: S2 Table — (DOCX) [file pbio.3000582.s009.docx]

**S2 Table. Primers used for ChIP-qPCR and RT-qPCR**

| Name | Sequence |
| --- | --- |
| 354 nt-p2-F  354 nt-p2-R | 5'TCCCGAATCATGTGCCCAAAC 3'  5'TTCGGTACGTGTCCCCTTTTCG 3' |
| 253-p3-F | 5'GGGCAAGGATAATTTCTCTACCAACGCTG 3' |
| 253-p3-R | 5'ATGCAGAAAATGAGGAAGTCCACGTTGAT 3' |
| Left 300 bp-p1-F  Left 300 bp-p1-R | 5'GCATGCTCTTTGTTATTAGGTCGAC 3'  5'CATCAAAATCAGCACGTGTAGCAAGTAAA 3' |
| Right 300 bp-p1-F | 5'GGGTATGCTTCGTTGTGGTTTAGTTGAG 3' |
| Right 300 bp-p1-F | 5'GGACCCCGGCCTGCAAGAGAGT 3' |
| Actin-F | 5'CCCGATTGAGCATGGCATTG 3' |
| Actin-R | 5'AGGTCACGCCCCGCAAGATC 3' |
| CentC-F | 5'TTCGGTACGTGTCCCCTTTTCG 3' |
| CentC-R | 5'GCATGTTCGTTGTGAAAAACG 3' |
| CRM2-LTR32-F | 5'TTGGAATGTTCAAGCACAACATGGAA 3' |
| CRM2-LTR32-R | 5'GCAAGTAGCGAGAGCTAAACTTGA 3' |
