# Supplementary material for: Back-spliced RNA from retrotransposon binds to centromere and regulates centromeric chromatin loops in maize
Source: PLoS Biol. 2020 Jan 29;18(1):e3000582. doi: 10.1371/journal.pbio.3000582 (PMC7010299; doi:10.1371/journal.pbio.3000582)
Supplement: S3 Table — (DOCX) [file pbio.3000582.s010.docx]

**S3 Table. Primers used for the full length detection of 354 nt RNA**

| Name | Sequence |
| --- | --- |
| Full-F1 | 5'CCGAACGTGTTACCTTTATTTTACCACCA 3' |
| Full-R1 | 5'TGGGTTCTTATTCTGATTATGCTGATTGC 3' |
| Full-F2 | 5'AGGCGTAGAAGGTGCAGGGCTG 3' |
| Full-R2 | 5'CCAGTGATTCTGAGGAAACTAGTC 3' |
| Full-F3 | 5'GGGACACGTACCGAACGTGTTACCTTTAT 3' |
| Full-R3 | 5'ATGCTATGATTGCCACTAACC 3' |
| Full-F4 | 5'CCGAACGTGTTACCTTTATTTTACCACCA 3' |
