# Supplementary material for: Back-spliced RNA from retrotransposon binds to centromere and regulates centromeric chromatin loops in maize
Source: PLoS Biol. 2020 Jan 29;18(1):e3000582. doi: 10.1371/journal.pbio.3000582 (PMC7010299; doi:10.1371/journal.pbio.3000582)
Supplement: S4 Table — (DOCX) [file pbio.3000582.s011.docx]

**S4 Table. Probes for RNA purification (with biotin labeled on the 3’ end of the probes)**

| Name | Sequence |
| --- | --- |
| Probe 1-60 (+) | 5'AAGAACCCAGCGAAAAGGGGACACGTACCGAACGTGTTACCTTTATTTTACCACCACATA 3' |
| Probe 1-60 (-) | 5'TATGTGGTGGTAAAATAAAGGTAACACGTTCGGTACGTGTCCCCTTTTCGCTGGGTTCTT 3' |
| Probe 121-180 (+) | 5'TGTGAAGAAGCGGCGCCTTTGGCAGGGGGCGCCACCGGCTTGGTCGTCCCTGTGCGCGAT 3' |
| Probe 121-180 (-) | 5'ATCGCGCACAGGGACGACCAAGCCGGTGGCGCCCCCTGCCAAAGGCGCCGCTTCTTCACA 3' |
| Probe 301-354 (+) | 5'ACTTCCTCATTTTCTGCATGGTTACTGGCAATCATAGCATGACTAGTTTCCTCAGA 3' |
| Probe 301-354 (-) | 5'TCTGAGGAAACTAGTCATGCTATGATTGCCACTAACCATGCAGAAAATGAGGAAGT 3' |
| Probe 244-294 (+) | 5'TTCTGATTATGCTGATTGCGATGTTATTCCAGCCCTGCACCTTCTACGCC 3' |
| Probe 244-294 (-) | 5'GGCGTAGAAGGTGCAGGGCTGGAATAACATCGCAATCAGCATAATCAGAA 3' |
