# Supplementary material for: Back-spliced RNA from retrotransposon binds to centromere and regulates centromeric chromatin loops in maize
Source: PLoS Biol. 2020 Jan 29;18(1):e3000582. doi: 10.1371/journal.pbio.3000582 (PMC7010299; doi:10.1371/journal.pbio.3000582)
Supplement: S5 Table — (DOCX) [file pbio.3000582.s012.docx]

**S5 Table. Probes for Northern blotting (with digoxin labeled on the 3’ end of the probes)**

| Name | Sequence |
| --- | --- |
| Probe (+) | 5'TGGCGCCCCCTGCCAAAGGCGCCGC 3' |
| Probe (-) | 5'GCGGCGCCTTTGGCAGGGGGCGCCA 3' |
