# Supplementary material for: Back-spliced RNA from retrotransposon binds to centromere and regulates centromeric chromatin loops in maize
Source: PLoS Biol. 2020 Jan 29;18(1):e3000582. doi: 10.1371/journal.pbio.3000582 (PMC7010299; doi:10.1371/journal.pbio.3000582)
Supplement: S6 Table — (DOCX) [file pbio.3000582.s013.docx]

**S6 Table. Primers used for RNA: DNA hybrid detection**

| Name | Sequence |
| --- | --- |
| 607-right-F | 5'GGGTATGCTTCGTTGTGGTTTAGTTGAG 3' |
| 607-right-R | 5'GGACCCCGGCCTGCAAGAGAGT 3' |
| 607-left-F | 5'GCATGCTCTTTGTTATTAGGTCGAC 3' |
| 607-left-R | 5'CATCAAAATCAGCACGTGTAGCAAGTAAA 3' |
| 85-1-F | 5'TGATTTGAACGACCATGATGTAA 3' |
| 85-1-R | 5'CGTTGGTAGAGAAATTATCCTTG 3' |
| 253-1-F | 5'CGGATGTGGATGTGTGTGAGTGG 3' |
| 253-1-R | 5'CAGGTTGCCCAGGCCGAAAA 3' |
| 253-2-F | 5'TGGAATAGAGTGTGTCGCTGA 3' |
| 253-2-R | 5'ATGCTATGATTGCCACTAACC 3' |
| 253-3-F | 5'ACGGATGTGGATGTGTGCGAGTG 3' |
| 253-3-R | 5'CGACGCCGATAGGTATGAGAGTC 3' |
| 269-1-F | 5'AACGTGGACTTCCTCATTTTCTG 3' |
| 269-1-R | 5'GCAGGCCGGGGTCCAACACACAG 3' |
| 269-2-F | 5'AGGTACGCTTGTTTGGGCAGTC 3' |
| 269-2-R | 5'GCCCTGCACCTTCTACGCCTAACA 3' |
| rRNA-F | 5'ACGACTCTCGGCAACGGATATCTCG 3' |
| rRNA-R | 5'GTGACGCCCAGGCAGACGTGCCCTC 3' |
| Zm00001d007960-F | 5'GCTTTGTCTTTGGCTTCGGA 3' |
| Zm00001d007960-R | 5'CGGTTGCAGATTTGGAGAGG 3' |
