# Supplementary material for: Back-spliced RNA from retrotransposon binds to centromere and regulates centromeric chromatin loops in maize
Source: PLoS Biol. 2020 Jan 29;18(1):e3000582. doi: 10.1371/journal.pbio.3000582 (PMC7010299; doi:10.1371/journal.pbio.3000582)
Supplement: S7 Table — (DOCX) [file pbio.3000582.s014.docx]

**S7 Table. Primers used for detection of the T7 endonuclease I digested fragments**

| Name | Sequence |
| --- | --- |
| T7-p1-F | 5'TCGCGAGCATACAAGTGCATAAC 3' |
| T7-p1-R | 5'TGAGGATTATTACCAGGAGTTAC 3' |
| T7-p2-F | 5'GGGTATGCTTCGTTGTGGTTTAGTTGAG 3' |
| T7-p2-R | 5'CATCAAAATCAGCACGTGTAGCAAGTAAA 3' |
| T7-p3-F | 5'CGACCTAATAACAAAGAGCATG 3' |
| T7-p3-R | 5'CAGGATATACTTGATTATAAGG 3' |
