# Supplementary material for: Back-spliced RNA from retrotransposon binds to centromere and regulates centromeric chromatin loops in maize
Source: PLoS Biol. 2020 Jan 29;18(1):e3000582. doi: 10.1371/journal.pbio.3000582 (PMC7010299; doi:10.1371/journal.pbio.3000582)
Supplement: S8 Table — (DOCX) [file pbio.3000582.s015.docx]

**S8 Table. Primers used for 3C-PCR**

| Name | Sequence |
| --- | --- |
| 3C-first-F | 5'CATGCTCTTTGTTATTAGGTCGAC 3' |
| 3C-first-R | 5'GACCCCGGCCTGCAAGAGAGTTAGTAT3' |
| 3C-second-F | 5'CAGCCCTGCACCTTCTACGCCTA 3' |
| 3C-second-R | 5'CCCGGCCTGCAAGAGAGTTAGTATATGTC3' |
| 3C-third-F | 5'GCATGCTCTTTGTTATTAGGTCGAC 3' |
| 3C-third-R | 5'CCGAACGTGTTACCTTTATTTTA 3' |
