# Supplementary material for: Back-spliced RNA from retrotransposon binds to centromere and regulates centromeric chromatin loops in maize
Source: PLoS Biol. 2020 Jan 29;18(1):e3000582. doi: 10.1371/journal.pbio.3000582 (PMC7010299; doi:10.1371/journal.pbio.3000582)
Supplement: S9 Table — (DOCX) [file pbio.3000582.s016.docx]

**S9 Table. Primers used for 3C-qPCR**

| Name | Sequence |
| --- | --- |
| 3C-P1-F | 5'CTTCGTCTCATCTGGATTTGTAC 3' |
| 3C-P2-F | 5'AACAAGCATTCGCGAGCATACAAGT 3' |
| 3C-P3-F | 5'TAGGTGTGGCTGATGTCGATTTGAC 3' |
| 3C-P4-F | 5'GCACAACAAGACTCTCATACCTATC 3' |
| 3C-P5-F | 5'GTCCCTGTGCGCGATGTAGTGCTAG 3' |
| 3C-P6-F | 5'TCACTAATGGCAGCCTTCACTTG 3' |
| 3C-P7-F | 5'TTAGGGGGATGACGAACCACACG 3' |
| 3C-P8-F | 5'TGTGGTCAGTGCATCAAGGCGTG 3' |
| 3C-P9-F | 5'ACTGCTCACAAGGCGCTCACACT 3' |
| 3C-P10-F | 5'TCGAACCCCAAACAGACCGTAGG 3' |
| 3C-P11-F | 5'GTGCGAGGCTCAAAAGGGTGCTG 3' |
| 3C-P12-F | 5'TCACCTATCAACCTGGAAATCTG 3' |
| 3C-P13-F | 5'AATCGCTGAACCAACTCCCTGTG 3' |
| 3C-P14-F | 5'TGGGGCGTTCGCAGCATTAAGTG 3' |
| 3C-P15-F | 5'AGCCAAGGAGAAGAAGGGACAGG 3' |
| 3C-P16-F | 5'GCGACGAGACAAAGCATCAGCAA 3' |
| 3C-P17-F | 5'TCACTCCAAGCAAACGGCACATC 3' |
| 3C-1-R | 5'GTTGAGGATTATTACCAGGAGTTAC 3' |
| 3C-2-R | 5'GCGTTTTCGTCGTGGTTTGAACAGG 3' |
| sam-DNA-P4-F | 5'AGATGGTCACGGCAACGGAAAACTC 3' |
| sam-DNA-P4-R | 5'GGATTTCTTTTCTGACACTTTGATTC 3' |
| sam-DNA-P7-F | 5'CCTCGTAACTGGCGTAGCTGAAACC 3' |
| sam-DNA-P7-R | 5'CTCCCTCCATCTCCAGCATTGTGTTC 3' |
