# Supplementary material for: Back-spliced RNA from retrotransposon binds to centromere and regulates centromeric chromatin loops in maize
Source: PLoS Biol. 2020 Jan 29;18(1):e3000582. doi: 10.1371/journal.pbio.3000582 (PMC7010299; doi:10.1371/journal.pbio.3000582)
Supplement: S10 Table — (DOCX) [file pbio.3000582.s017.docx]

**S10 Table. Primers used for RNAi plasmid constructing**

| Name | Sequence |
| --- | --- |
| 289 bp-XhoI+BglII-F | 5'CCGCTCGAGTGAAGAGTACTCACC 3' |
| 289 bp-XhoI+BglII-R | 5'GGAAGATCTTGTGGTGGTAAAAT 3' |
