# Supplementary material for: Back-spliced RNA from retrotransposon binds to centromere and regulates centromeric chromatin loops in maize
Source: PLoS Biol. 2020 Jan 29;18(1):e3000582. doi: 10.1371/journal.pbio.3000582 (PMC7010299; doi:10.1371/journal.pbio.3000582)
Supplement: S11 Table — (DOCX) [file pbio.3000582.s018.docx]

**S11 Table. Primers used for cloning 1671 bp CRM1 into pET-30a vector**

| Name | Sequence |
| --- | --- |
| CRM1-1660 bp-p3-F | 5'AGAAAATAACGGCACCACTAAG 3' |
| CRM1-1660 bp-p3-R | 5'GCAGGAATACGGCGGTGACACT 3' |
| 1671 bp-p1-F | 5'GATCTCCTTCGTCTCATCTGG 3' |
| 1671 bp-p1-R | 5'TGGGTTCTTATTCTGATTATGAATTCGCTGATTGC  GATGTTATTCC 3' |
| 1671 bp-p2-F | 5'GAATTCATAATCAGAATAAGAACCCAG 3' |
| 1671 bp-p2-R | 5'GATCCTGCTGCATATCTTGATTG 3' |
| 1671 bp-p3-F | 5'GATCTCCTTCGTCTCATCTGG 3' |
| 1671 bp-p3-R | 5'GGATCCGCCAGTGGCGCCCCCTGCCAA 3' |
| 1671 bp-p4-F | 5'TGGCAGGGGGCGCCACTGGCGGATCCTTGGTC  GTCCCTGTGCGCG 3' |
| 1671 bp-p4-R | 5'GATCCTGCTGCATATCTTGATTG 3' |
| 1671 bp-XbaI+HindIII-F | 5'GCTCTAGAGATCTCCTTCGTCTCATCTGG 3' |
| 1671 bp-XbaI+HindIII-R | 5'CCCAAGCTTGATCCTGCTGCATATCTTGATTG 3' |
| 1671 bp-HindIII+XbaI-F | 5'CCCAAGCTTGATCTCCTTCGTCTCATCTGG 3' |
| 1671 bp-HindIII+XbaI-R | 5'GCTCTAGAGATCCTGCTGCATATCTTGATTG 3' |
